# Sustainable EV adoption with clustering and predictive modelling for optimal charging infrastructure in the West Midlands and North East UK

**DOI:** 10.1038/s41598-026-43106-6

**Published:** 2026-05-07

**Authors:** Muhammed Cavus, Shouai Wang, Sanchari Deb, Anurag Sharma, Margaret Bell, Dilum Dissanayake

**Affiliations:** 1 School of Engineering Physics and Mathematics, Northumbria University, Newcastle Upon Tyne, NE1 8SA UK; 2 Faculty of Engineering and Natural Sciences, Iskenderun Technical University, Hatay, 31200 Türkiye; 3 Department of Engineering, Durham University, Durham, DH1 3LE UK; 4 Institute of Hazard, Risk and Resilience (IHRR), Durham University, Durham, DH1 3LE UK; 5 School of Engineering, Newcastle University, Newcastle Upon Tyne, NE1 7RU UK; 6 Faculty of Science, Agriculture & Engineering (SAgE), Newcastle University, Newcastle Upon Tyne, NE1 7RU UK; 7 School of Geography, Earth and Environmental Sciences, University of Birmingham, Birmingham, B15 2TT UK

**Keywords:** Electric vehicles, Smart charging, Clustering, Predictive modelling, Machine learning, Sustainable mobility, ISE-CAP, Environmental impact, Socioeconomic scenarios, Sustainability, Energy grids and networks

## Abstract

The rapid growth of electric vehicle (EV) adoption presents significant challenges for charging infrastructure planning and grid integration, particularly at the regional level. However, existing studies often apply machine learning techniques in isolation and lack integrated, region-specific behavioural modelling. This study introduces the Intelligent Sustainable EV Clustering and Analysis Platform (ISE-CAP), an integrated and interpretable analytical framework that advances beyond conventional ML-based EV studies by combining behavioural clustering, predictive modelling, explainable artificial intelligence (XAI), and adaptive optimisation within a regionally comparative decision-support architecture. A structured survey dataset comprising 256 EV users from the North East (n = 124) and West Midlands (n = 132) was analysed to examine charging behaviour, adoption motivations, and infrastructure preferences. K-Means clustering identified three distinct EV user groups in each region. Predictive models, including Random Forest, CatBoost, and a compact deep learning architecture, were trained using an 80:20 train-test split with cross-validation achieved high accuracy on the available regional dataset, with the North East model attaining $\hbox {R}^{2}$ = 0.9951 and MSE = 0.0694, indicating a very strong fit to the observed data. On the held-out regional test dataset, the West Midlands model achieved $\hbox {R}^{2}$= 0.9548 and MSE = 0.6410, indicating strong predictive performance within the analysed sample. Charging behaviour analysis indicates that 85% of users prefer DC fast chargers, with most users (66% in the West Midlands and 53% in the North East) willing to travel up to 3 km for charging. Cost savings accounted for 65% of EV purchases in the North East, while environmental concerns accounted for 30% in the West Midlands, based on regional frequency distributions. SHapley Additive exPlanations (SHAP) analysis identified charging duration, real-time station availability, and cost as the most influential factors associated with charger selection preferences. The findings highlight regional heterogeneity in charging behaviour and infrastructure needs, emphasising the importance of adaptive and interpretable modelling approaches. While results are specific to the analysed regions, the ISE-CAP framework provides a scalable decision-support architecture for sustainable EV infrastructure optimisation in smart city contexts.

## Introduction

### Introduction and literature review

The rapid global adoption of electric vehicles (EVs) has been driven by the increasing need for sustainable transportation and the urgent demand for reducing carbon emissions^1^. Governments worldwide are implementing policies and incentives to promote EV adoption, yet challenges remain in ensuring efficient charging infrastructure and optimising energy management^2^. The transition to EVs requires robust planning and data-driven strategies to align charging networks with user demand while minimising grid stress and travel inconvenience^3^.

In the UK, the EV market has experienced significant growth, with sales increasing by over 40% in recent years^4^. However, regional disparities in EV adoption and infrastructure availability remain a key concern^5^. The North East and West Midlands represent two distinct regions with varying levels of EV uptake, charging behaviour, and accessibility to public and private charging networks^6^. Understanding these regional differences is critical for improving infrastructure planning and developing tailored policies to encourage widespread EV adoption^7,8^. According to the UK Department for Transport, battery EV registrations have increased by over 40% year-on-year in recent reporting periods, with EVs accounting for a rapidly growing share of new vehicle sales. Concurrently, public charging infrastructure expansion has struggled to match projected demand growth, reinforcing concerns regarding grid stress, accessibility disparities, and infrastructure readiness. International Energy Agency reports similarly highlight that charging infrastructure deployment must accelerate significantly to meet net-zero transport targets. These quantitative trends underscore the urgency of data-driven infrastructure optimisation^5,8^.

Recent advances in urban mode choice modelling further contextualise the behavioural component of this study. For example, Ranjan and Sinha^9^ employed a Multinomial Logit (MNL) framework to analyse work-trip mode selection in urban environments, demonstrating the significant influence of travel time, comfort, safety, and cost attributes. Similarly, subsequent work on commuter satisfaction and service efficiency^10^ applied Importance–Performance Analysis (IPA) combined with regression modelling to capture demographic heterogeneity in transport evaluation.

While these discrete choice and regression-based approaches provide strong interpretability and behavioural grounding, they are typically static and do not integrate predictive learning or adaptive optimisation layers. The proposed Intelligent Sustainable EV Clustering and Analysis Platform (ISE-CAP) framework extends beyond traditional MNL-based behavioural modelling by embedding clustering, nonlinear prediction, explainable feature attribution (SHAP), and reinforcement learning within a unified architecture. This enables dynamic infrastructure optimisation informed by behavioural segmentation rather than isolated utility estimation.

#### Novelty and distinction from prior studies

While previous studies have applied individual machine learning (ML) techniques such as clustering, deep learning (DL), reinforcement learning (RL), or SHapley Additive exPlanations (SHAP)-based feature interpretation to EV behaviour analysis, the proposed ISE-CAP advances the literature through a fully integrated and adaptive analytical architecture^2,5,8,11^.

First, unlike conventional approaches that apply clustering or prediction models in isolation, ISE-CAP combines behavioural clustering, predictive modelling, explainable artificial intelligence (XAI), and adaptive optimisation within a unified framework. This integration enables not only behavioural segmentation and demand forecasting, but also dynamic infrastructure strategy refinement based on interpretable model feedback. Second, the framework explicitly incorporates region-specific modelling by comparatively analysing two socio-economically distinct UK regions. Rather than developing a generic predictive model, ISE-CAP demonstrates how regional heterogeneity influences model performance, behavioural clustering patterns, and infrastructure requirements. This comparative regional modelling provides practical insights for geographically tailored policy interventions.

Third, ISE-CAP uniquely integrates structured behavioural survey data with predictive ML techniques. Many prior studies rely solely on charging session logs or grid-level data. In contrast, this framework embeds user motivations, travel behaviour, socio-economic attributes, and perception-based variables directly into the modelling pipeline, enabling a more user-centric infrastructure optimisation strategy. Fourth, the inclusion of a RL–based optimisation component introduces adaptive charging station placement strategies informed by predicted demand, user travel distance, and grid impact. This extends beyond static infrastructure planning models commonly reported in the literature.

Finally, the use of SHAP-based explainability within a feedback loop ensures that policy recommendations are derived from transparent and quantifiable feature contributions. This interpretability layer bridges the gap between advanced ML outputs and actionable policy design, which remains underdeveloped in many EV infrastructure studies. Collectively, these contributions position ISE-CAP not merely as a combination of existing methods, but as a structured, interpretable, and regionally adaptive decision-support framework for sustainable EV infrastructure planning in smart cities.

Despite significant advancements in EV charging research, several limitations remain in the existing literature. First, many studies apply clustering, predictive modelling, or optimisation techniques in isolation, without integrating them into a unified analytical framework. Second, behavioural survey data capturing user motivations, socio-economic attributes, and perception-based factors are often analysed descriptively rather than embedded within predictive modelling pipelines. Third, region-specific comparative analyses within the UK context remain limited, reducing the ability to design geographically tailored infrastructure strategies. This study addresses these gaps by developing the ISE-CAP framework, which integrates behavioural clustering, predictive modelling, XAI, and adaptive optimisation within a comparative regional architecture.

The increasing penetration of EVs has necessitated extensive research on charging infrastructure, user behaviour, and predictive modelling. Previous studies have examined the adoption of EVs from technological, economic, and environmental perspectives, highlighting the need for optimised charging networks to support long-term sustainability^12–16^.

#### EV adoption and charging infrastructure

The adoption of EVs is influenced by multiple factors, including financial incentives, environmental consciousness, and charging infrastructure availability. Government subsidies, lower operational costs, and tax reductions have significantly accelerated EV adoption in the United Kingdom^1^. Financial incentives, such as grants and rebates, have been instrumental in increasing EV registrations, particularly in urban regions where policy interventions are more prevalent^17,18^. However, disparities remain, as lower-income groups often struggle with the high upfront cost of EVs despite available incentives^3^.

Beyond financial considerations, charging infrastructure plays a fundamental role in influencing EV adoption rates. While urban areas have benefited from increasing numbers of charging stations, suburban and rural locations still face significant challenges due to inadequate charging accessibility^6^. The UK has made considerable progress in expanding public charging networks, yet gaps persist, particularly in remote areas where home charging is less feasible^4^. Research suggests that improving the spatial distribution of charging stations and prioritising high-traffic areas can mitigate range anxiety and enhance EV uptake^19^.

Recent studies have advanced the optimisation of charging infrastructure placement using demand-driven modelling approaches. For instance, Hamed et al. (2023) employed a Maximum Covering Location Model (MCLM) combined with a random parameters framework to optimise EV charging facility locations based on anticipated charging demand, charging technologies, and spatial coverage constraints. Their mixed-integer optimisation approach integrates financial feasibility and power demand heterogeneity, offering a rigorous optimisation perspective for metropolitan infrastructure deployment. While such models provide strong spatial optimisation capability, they primarily focus on aggregate demand estimation rather than integrating individual-level behavioural segmentation and explainable predictive analytics within a unified decision-support framework^20^.

Additionally, Hamed et al. (2021) examined household satisfaction with the first EV and its influence on the intention to purchase a second EV using random parameters ordered probit models. Their findings demonstrate that satisfaction levels, charging methods, running cost savings, vehicle range, and brand attributes significantly influence repurchase timing. This behavioural perspective underscores the importance of user experience and perceived performance in shaping long-term EV adoption trajectories. The present study complements this line of research by embedding perception-based and socio-economic attributes directly within ML models to examine how behavioural and satisfaction-related factors are associated with charging preferences and infrastructure utilisation patterns^21^.

Environmental consciousness has also been a key driver in EV adoption. Recent studies indicate that consumer awareness regarding carbon emissions and sustainability is increasingly shaping purchasing decisions, with a growing number of buyers opting for EVs due to environmental concerns^22^. However, a more comprehensive approach is needed to integrate financial incentives, infrastructure development, and public awareness campaigns to sustain this momentum.

Despite these advancements, challenges remain in ensuring the equitable roll-out of EV infrastructure across different socio-economic groups. Policymakers must address charging availability and affordability disparities to facilitate a smoother transition toward widespread EV adoption in the UK^3^.

#### ML for EV behaviour analysis

ML has been used to analyse EV user behaviour and predict charging demand, improving infrastructure planning and efficiency^23,24^. Clustering techniques such as K-Means, Density-Based Spatial Clustering of Applications with Noise (DBSCAN), and hierarchical clustering have been employed to segment EV users based on travel patterns, charging frequency, and socio-economic factors^23^. These clustering models assist in optimising charging station placement by identifying high-density user clusters and forecasting demand patterns in urban and suburban areas^24^.

DL models, including artificial neural networks (ANNs), recurrent neural networks (RNNs), and long short-term memory (LSTM) networks, have demonstrated high accuracy in predicting charging demand. A study by Yaghoubi et al.^23^ reviewed various ML and DL approaches for EV charging prediction, demonstrating the effectiveness of ensemble models in improving accuracy. Similarly, a hybrid model integrating support vector machines (SVM) and deep neural networks (DNN) achieved a 95% accuracy rate in forecasting EV charging demand^25^.

Feature importance analysis has provided critical insights into EV charging preferences. SHAP analysis has been particularly effective in identifying key determinants influencing charging behaviour. Recent studies highlight that charging duration, station proximity, electricity pricing, and real-time availability significantly impact user decisions^26^. Additionally, dynamic pricing strategies have been explored to encourage off-peak charging, reducing grid congestion and enhancing overall charging infrastructure efficiency^11^. Recent studies have also explored the application of RL in optimising EV charging strategies^27^. Chen et al.^25^ developed an RL-based algorithm to optimise charging station operations, reducing peak demand fluctuations by 18%. Similarly, Wang et al.^26^ proposed an RL-based scheduling system for EV clusters, demonstrating its ability to balance electricity demand and prevent grid overloading.

Recent studies on behavioural transport modelling have emphasised the importance of socio-demographic heterogeneity and value-of-time estimation in shaping commuter decisions. For instance, Ranjan and Sinha^28^ utilised a MNL model to estimate value of travel time (VOT) across income and gender groups, highlighting the necessity of disaggregated behavioural modelling in mid-sized urban contexts. Similarly, the influence of integrated multimodal travel information on commuter preferences^29^ demonstrates how real-time information systems can significantly alter modal shift probabilities, reinforcing the behavioural impact of digital infrastructure. A recent systematic review of mode choice behaviour^30^ further synthesises determinants across demographic, trip-specific, and infrastructural dimensions, identifying the need for integrated analytical frameworks capable of capturing multidimensional interactions. These findings align with the motivation of the present study, which embeds socio-economic attributes, perception-based variables, and charging behaviour indicators directly into ML pipelines.

Moreover, emerging optimisation-focused studies in transport infrastructure^10^ underscore the importance of combining behavioural modelling with performance-based evaluation metrics. However, most existing frameworks rely on either static regression or discrete choice structures without incorporating adaptive optimisation mechanisms. The ISE-CAP framework addresses this gap by integrating behavioural segmentation, nonlinear prediction, explainable AI, and simulation-based RL within a cohesive decision-support architecture.

Table 1 presents a comparative analysis of traditional EV charging methods, ML-based approaches, and our proposed ISE-CAP framework. Traditional methods rely on static models and rule-based charging management, making them less adaptive to real-time demand variations^31^. ML-based approaches introduce predictive capabilities using LSTM models and clustering techniques, improving demand forecasting and infrastructure planning^25^. However, these methods often lack dynamic adaptation for station placement and grid load balancing. The proposed ISE-CAP method combines LSTM with RL, allowing real-time charging demand prediction and adaptive station optimisation^32^. Additionally, ISE-CAP incorporates SHAP-based feature importance analysis to refine infrastructure planning and policy recommendations^33^. This framework extends conventional modelling approaches by integrating behavioural segmentation, explainability, and adaptive optimisation within a unified architecture. While not intended to replace traditional methods, ISE-CAP provides complementary analytical capabilities for region-specific infrastructure planning.

**Table 1:** Comparison of traditional, ML, and ISE-CAP methods for EV charging.

Feature	Traditional methods	Other ML methods	ISE-CAP method (proposed)
Charging Demand Prediction	Static models, low adaptability^31^	LSTM, DL with moderate accuracy^25^	LSTM with RL for real-time demand prediction^25^
Infrastructure Planning	Fixed locations, demand estimation from surveys^34^	Clustering methods such as K-Means and DBSCAN^25^	Hybrid clustering with dynamic adjustments^34^
Charging Load Management	Rule-based or time-of-use pricing^35^	RL-based schedule adjustment^36^	Adaptive RL model for optimal grid load balancing^32^
Feature Importance Analysis	Limited interpretability, predefined parameters^37^	Uses SHAP but lacks real-time adaptation^33^	Real-time SHAP feature analysis with feedback loop^33^
User Behaviour Modelling	Manual surveys, predefined rules^38^	Predictive analytics, lacks dynamic clustering^37^	Intelligent clustering with demand forecasting^37^
Charging Station Optimisation	No optimisation, static planning^39^	Basic RL for placement^37^	Advanced RL with continuous optimisation^39^
Policy and Infrastructure Recommendations	General policies, not data-driven^31^	Policy recommendations based on fixed models^35^	Dynamic policy recommendations with scenario simulations^35^

These advancements in ML and DL are instrumental in improving EV infrastructure planning, enabling predictive modelling for better decision-making, and ensuring the efficient integration of EVs into the energy grid.

#### Comparative studies on regional EV adoption

Regional disparities in EV adoption have been widely studied, particularly in terms of socio-economic and infrastructural differences^40–42^. A study by Nieto et al.^43^ assessed the socio-economic impact of EV adoption in the UK, highlighting that regional imbalances in charging infrastructure have contributed to uneven adoption rates. Similarly, Arslangulova and Galanakis^44^ investigated public EV charging accessibility in Nottingham and Frankfurt, demonstrating that urban users rely more on public charging infrastructure, whereas suburban users prefer home charging solutions due to greater residential parking availability.

Furthermore, regional policies and incentives play a significant role in adoption rates. A study by Hopkins et al.^3^ examined government subsidies and their impact on EV adoption, revealing that areas with targeted incentives and better infrastructure experience faster uptake. Similarly, Caulfield et al.^45^ explored the equity impacts of EV subsidies, finding that lower-income groups often face barriers to EV adoption despite financial incentives.

In a broader European context, Xue et al.^46^ analysed EV adoption trends in Germany, France, and the UK, highlighting disparities in public investment and charging infrastructure accessibility. Their findings suggest that regions with higher investment in fast-charging networks exhibit faster EV adoption rates. Similarly, a study by Haidar and Rojas^47^ found that Norway’s extensive charging infrastructure and tax incentives contributed to the highest EV adoption rate in Europe, demonstrating the impact of policy-driven initiatives on regional adoption patterns.

Moreover, income levels and education significantly influence EV adoption trends. Mukherjee^48^ developed a framework to measure regional disparities in EV adoption in Ireland, revealing that high-income households are more likely to transition to EVs, while lower-income groups express concerns over affordability and charging accessibility. This underscores the need for targeted policy interventions to address socio-economic disparities in EV adoption.

While prior studies demonstrate strong performance in isolated clustering or predictive modelling tasks, several limitations remain evident. Many clustering studies rely solely on travel logs without integrating socio-economic or perception-based variables, reducing behavioural depth. Predictive models frequently prioritise accuracy without incorporating explainability mechanisms, limiting policy interpretability. Additionally, most frameworks lack adaptive optimisation layers capable of translating predictions into infrastructure planning strategies. The proposed ISE-CAP framework addresses these limitations by integrating behavioural clustering, predictive modelling, SHAP-based explainability, and RL–based optimisation within a unified architecture, thereby bridging the gap between analytical modelling and actionable infrastructure policy.

By integrating clustering analysis, predictive modelling, and feature importance evaluation, this study builds upon existing research to comprehensively assess EV user behaviour. The ISE-CAP framework offers a novel approach to analysing regional EV trends and infrastructure optimisation, contributing valuable insights for policymakers and urban planners.

#### Research questions

This study addresses the following research questions:

- What are the primary demographic, behavioural, and motivational differences in EV adoption between the North East and West Midlands regions?
- How can ML techniques classify EV users based on their charging habits and travel patterns?
- What are the most influential factors affecting EV charging preferences, and how do they vary between the two regions?
- How accurately can predictive models forecast EV charging behaviours, and what are the key performance differences between the North East and West Midlands?
- What strategies can be implemented to enhance EV infrastructure, improve user satisfaction, and support sustainable mobility?

#### Research gap, novelty, and contributions

Existing studies on EV charging behaviour and infrastructure planning frequently apply clustering, predictive modelling, or optimisation techniques independently. While discrete choice models and regression-based approaches provide behavioural interpretability, they often lack adaptive optimisation capability. Conversely, ML models may achieve high predictive accuracy but frequently omit explainability and policy translation layers.

This study addresses these gaps by introducing the ISE-CAP, an integrated analytical architecture that combines behavioural clustering, nonlinear predictive modelling, XAI, and simulation-based RL. The framework enables (i) behavioural segmentation, (ii) demand forecasting, (iii) feature-level interpretability, and (iv) adaptive infrastructure optimisation within a unified decision-support pipeline.

The key contributions are: (1) region-specific behavioural modelling across two socio-economically distinct UK regions; (2) integration of perception-based survey variables into predictive ML architectures; (3) SHAP-based feature attribution for policy interpretability; and (4) a simulation-based RL optimisation module for adaptive station placement. This structured integration distinguishes ISE-CAP from isolated modelling approaches reported in prior literature.

#### Organisation of the paper

This paper is structured into five sections. The introduction introduces the study’s background and motivation, highlighting the challenges associated with EV adoption and charging infrastructure in the context of sustainable mobility. It also emphasises the importance of data-driven analysis in optimising EV infrastructure. The literature review section presents a comprehensive review of relevant literature, discussing previous studies on EV user behaviour, clustering methodologies, predictive modelling, and key factors influencing charging preferences and infrastructure planning. The methodology section outlines the methods employed in this research, detailing the data collection process, clustering techniques, predictive models, and feature importance analysis using SHAP and decision trees. The results and discussion section presents the results, including identifying regional clustering patterns, evaluating predictive models’ performance, and analysing influential factors shaping EV user preferences. Finally, the conclusion section concludes the study by summarising the key research contributions, discussing policy implications, and suggesting potential directions for future research in optimising EV infrastructure and smart city development. This structured approach ensures a comprehensive understanding of EV user behaviours and charging infrastructure, contributing valuable insights for advancing sustainable urban mobility.

## Methodology

This study leverages the ISE-CAP framework, a comprehensive methodology designed to analyse EV user behaviours, predict charging preferences, and identify influential factors affecting charging station selection, user satisfaction, and infrastructure optimisation. This study provides actionable insights into optimising EV infrastructure across the North East and the West Midlands by integrating clustering, predictive modelling, and feature importance analysis using state-of-the-art ML techniques. The methodological components are outlined in detail below.

### Methodological rationale and framework linkages

The development of the ISE-CAP framework is motivated by the need to bridge behavioural analysis and infrastructure optimisation within a unified analytical pipeline. Existing EV studies frequently apply clustering, predictive modelling, or optimisation independently, limiting their ability to translate behavioural insights into adaptive infrastructure planning.

The rationale for integration in this study is structured as follows: First, behavioural clustering is employed to identify distinct EV user segments based on charging habits, socio-economic attributes, and travel patterns. This segmentation establishes the heterogeneity structure within each region.Second, predictive modelling is applied to estimate charging behaviour outcomes, enabling quantification of demand variability and behavioural intensity within and across clusters.

Third, XAI is incorporated to interpret model outputs and identify the most influential features associated with charging preferences. This step enhances policy interpretability by translating model predictions into understandable behavioural drivers. Fourth, simulation-based RL is introduced as an optimisation layer that utilises predicted demand and behavioural patterns to evaluate adaptive charging station placement strategies. The integrated structure ensures that clustering informs prediction, prediction informs explainability, and explainability informs optimisation. This sequential linkage establishes a coherent decision-support framework rather than a collection of independent modelling exercises.

### Data analysis

Table 2 provides a detailed demographic comparison between the North East and West Midlands, covering key categories such as gender, age distribution, occupation, education level, and annual household income. Descriptive percentages reported for EV adoption motivations were computed using frequency distributions within each regional sample. To assess whether regional differences in adoption drivers were statistically significant, chi-square tests of independence were conducted. Differences were considered statistically significant at p < 0.05.

In terms of gender, the North East has a higher proportion of males (56.5%) compared to the West Midlands (48.5%), whereas the West Midlands has a greater percentage of females (51.5%). Non-binary representation remains minimal in both regions.

**Table 2:** Comparison of demographic counts and percentages for North East and West Midlands.

North East (124)	West Midlands (132)
Gender	
Male: 70 (56.5%)	Male: 64 (48.5%)
Female: 53 (42.7%)	Female: 68 (51.5%)
Non-binary: 1 (0.8%)	Non-binary: -
Age	
18–24: 16 (12.9%)	18–24: 17 (12.9%)
25–34: 46 (37.1%)	25–34: 55 (41.7%)
35–44: 39 (31.5%)	35–44: 28 (21.2%)
45–54: 18 (14.5%)	45–54: 22 (16.7%)
55–64: 4 (3.2%)	55–64: 8 (6.1%)
65+: 1 (0.8%)	65+: 2 (1.5%)
Occupation	
Employed: 106 (85.5%)	Employed: 108 (81.8%)
Retired: 3 (2.4%)	Retired: 2 (1.5%)
Self-employed: 5 (4.0%)	Self-employed: 8 (6.1%)
Student: 6 (4.8%)	Student: 9 (6.8%)
Unemployed: 4 (3.2%)	Unemployed: 5 (3.8%)
Education	
Less than high school: 0 (0.0%)	Less than high school: 1 (0.8%)
High school diploma: 10 (8.1%)	High school diploma: 12 (9.1%)
Some college: 19 (15.3%)	Some college: 29 (22.0%)
Bachelor’s degree: 68 (54.8%)	Bachelor’s degree: 62 (47.0%)
Master’s degree: 25 (20.2%)	Master’s degree: 27 (20.5%)
Doctorate or higher: 2 (1.6%)	Doctorate or higher: 1 (0.8%)
Annual Household Income	
Less than$\pounds$20,000: 2 (1.6%)	Less than$\pounds$20,000: 6 (4.5%)
$\pounds$20,000-$\pounds$35,000: 30 (24.2%)	$\pounds$20,000-$\pounds$35,000: 17 (12.9%)
$\pounds$35,001-$\pounds$50,000: 28 (22.6%)	$\pounds$35,001-$\pounds$50,000: 17 (12.9%)
$\pounds$50,001-$\pounds$70,000: 35 (28.2%)	$\pounds$50,001-$\pounds$70,000: 28 (21.2%)
Over$\pounds$70,000: 29 (23.4%)	Over$\pounds$70,000: 64 (48.5%)

The age distribution highlights that the West Midlands has a higher proportion of individuals aged 25–34 (41.7%), compared to 37.1% in the North East. The North East has a greater share of individuals aged 35–44 (31.5%) than the West Midlands (21.2%). The proportion of those aged 45 and above is relatively low in both regions, with the highest representation being in the 45–54 range (14.5% in the North East and 16.7% in the West Midlands). Occupationally, most respondents in both regions are employed, with the North East reporting a higher employment rate (85.5%) compared to the West Midlands (81.8%). Self-employment is slightly more prevalent in the West Midlands (6.1%) than in the North East (4.0%). Retirement and unemployment rates remain low, with the North East having 2.4% retired individuals compared to 1.5% in the West Midlands.

Regarding education, a larger proportion of individuals in the North East hold a bachelor’s degree (54.8%) compared to the West Midlands (47.0%). Master’s degree holders are similarly distributed in both regions (20.2% in the North East and 20.5% in the West Midlands), while those with doctorate-level education are relatively rare. Income distribution varies significantly between the two regions. In the North East, 28.2% of households fall within the $\pounds$50,001–$\pounds$70,000 bracket, whereas in the West Midlands, a significant 48.5% earn over $\pounds$70,000, showing a notable income disparity. Conversely, lower-income brackets (less than $\pounds$35,000) are more prevalent in the North East compared to the West Midlands The majority of respondents reported owning a single EV at the time of participation. The survey instrument focused primarily on charging behaviour, infrastructure utilisation, and adoption motivations, and did not systematically model the intention to purchase a second EV as a core analytical variable. Future research may extend this framework by incorporating second-EV adoption intention and longitudinal ownership dynamics.

**Fig. 1:**
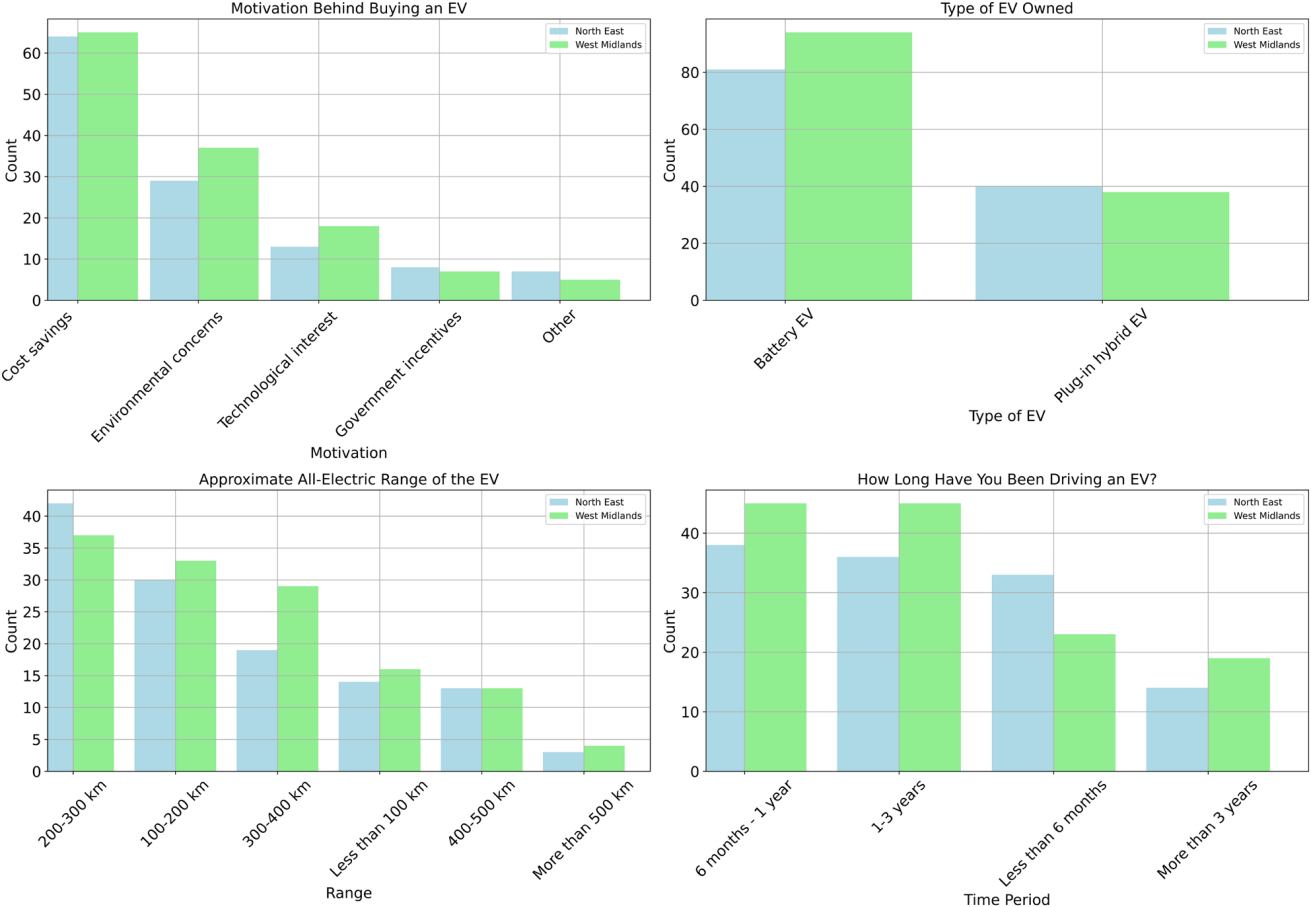
Comparison of EV ownership factors between North East and West Midlands.

**Fig. 2:**
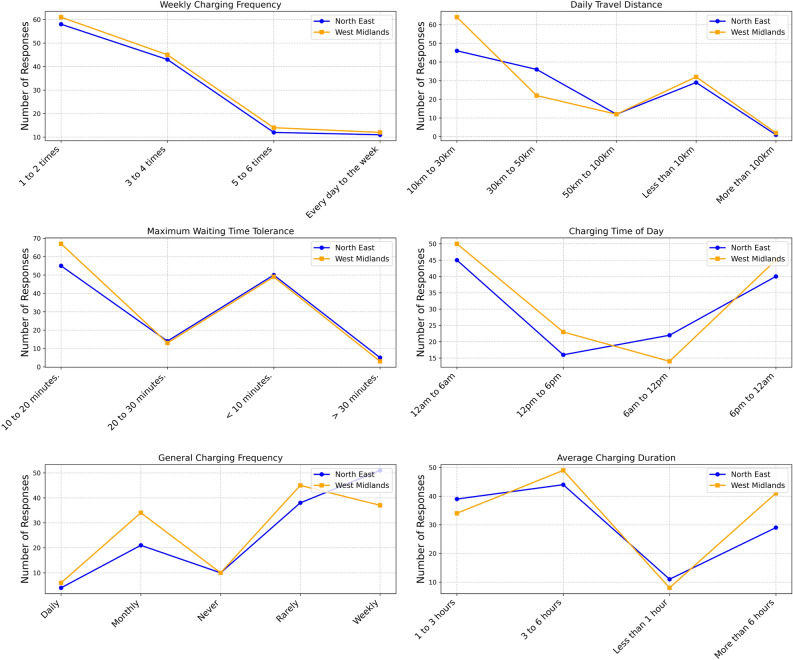
Comparison of EV charging behaviour and travel patterns between the North East and the West Midlands.

**Fig. 3:**
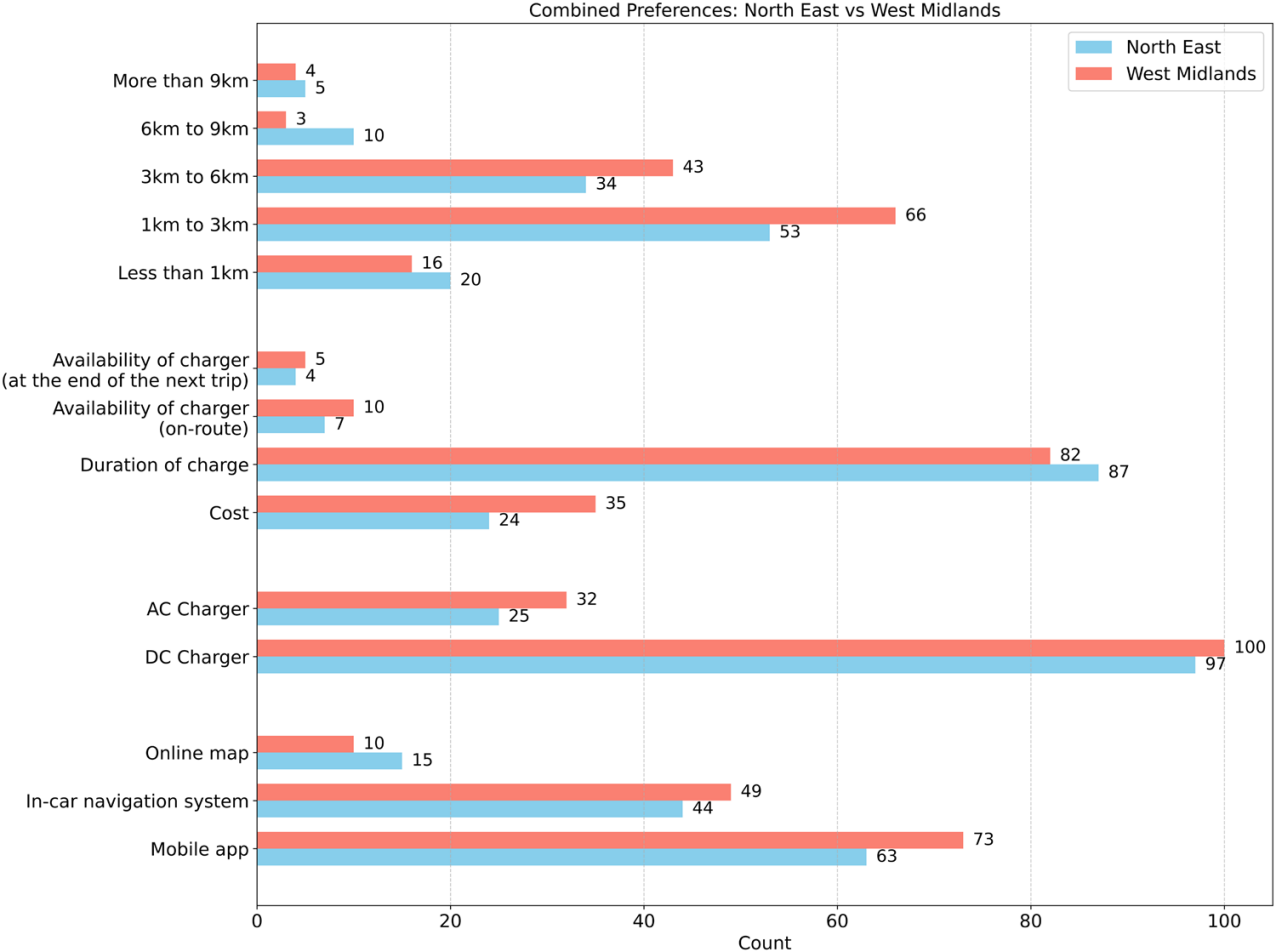
Comparison of EV charging preferences between the North East and West Midlands.

Chi-square tests of independence were conducted to evaluate regional differences in EV adoption motivations and charging preferences. For adoption motivation, the association between region and primary motivation was statistically significant ($\chi ^{2}(4) = 12.87$, $p = 0.012$), with a moderate effect size (Cramér’s $V = 0.224$). For charger type preference (AC vs. DC), no statistically significant regional difference was observed ($\chi ^{2}(1) = 1.94$, $p = 0.164$). Bootstrapped 95% confidence intervals were computed for proportional differences to quantify uncertainty. These results provide formal statistical support for the descriptive comparisons presented in Figures 1–3.

### Sampling strategy and representativeness

The study dataset consists of 256 EV users (North East: 124; West Midlands: 132) who participated in a structured survey designed to capture behavioural, socio-economic, and charging preference characteristics. Participants were eligible if they were current EV owners residing within the respective regions at the time of data collection. Respondents were recruited through a combination of regional EV user groups, university-affiliated outreach networks, local sustainability forums, and online community platforms focused on electric mobility. Participation was voluntary, and no financial incentives were provided. The sampling strategy therefore reflects a non-probability, self-selection approach. The dataset was not designed to be nationally representative of all UK EV users. Rather, it provides a structured behavioural sample from two specific regions. While certain demographic characteristics broadly align with regional EV ownership trends, the sample should be interpreted as regionally indicative rather than statistically representative of national EV registration distributions.

While this recruitment method enabled targeted engagement with active EV users, it may introduce self-selection bias, as individuals with stronger engagement in EV communities or higher digital accessibility may be overrepresented. As a result, the sample may not fully represent the broader regional EV population, particularly individuals with limited digital engagement or lower socio-economic accessibility. To assess potential confounding effects, socio-demographic variables including income level, education, occupation, age, and gender were incorporated into the clustering and predictive modelling pipeline. These variables were standardised and included as model features to account for behavioural variability linked to socio-economic differences. This approach mitigates confounding effects within the modelling process, although it does not eliminate sampling bias at the population level.

The income distribution indicates a relatively higher representation of upper-income households in the West Midlands sample, while mid-income groups are more prevalent in the North East. This imbalance reflects regional economic differences but may also influence charging behaviour patterns and model generalisability. Consequently, the findings should be interpreted as regionally indicative rather than statistically representative of the entire EV-owning population in the UK. Future research may benefit from stratified random sampling or integration with national EV registration datasets to enhance representativeness and external validity. Despite these limitations, the dataset provides a robust behavioural cross-section of active EV users in both regions and enables meaningful comparative modelling of regional charging patterns and infrastructure needs.

The North East and West Midlands were selected due to their contrasting urban structures, socio-economic profiles, and infrastructure distributions, providing analytically meaningful regional heterogeneity. The North East is characterised by a relatively compact urban layout with concentrated charging infrastructure, whereas the West Midlands encompasses metropolitan centres alongside suburban and rural areas with more dispersed travel patterns. Additionally, income distribution and mobility behaviours differ significantly between the two regions. These contrasts enable comparative modelling of homogeneous versus heterogeneous EV usage environments, enhancing policy relevance for region-specific infrastructure planning within the UK decarbonisation strategy.

The voluntary and community-based recruitment approach may introduce selection bias, as participants with stronger engagement in EV networks or higher digital accessibility may be overrepresented. Consequently, the sample may not fully represent the entire EV-owning population in each region. The findings should therefore be interpreted as behaviourally indicative within the sampled cohort rather than statistically representative of the broader UK EV population.

### Analysis of EV user motivations and preferences

Figure 1 presents a comparative analysis of EV ownership and usage patterns between respondents from the North East and the West Midlands regions. The four subplots highlight key aspects, including motivations for EV adoption, types of EVs owned, their approximate all-electric range, and the duration for which respondents have been driving an EV. A chi-square test was conducted to examine regional differences in BEV versus PHEV ownership. The association between region and vehicle type was not statistically significant ($\chi ^{2}(1) = 0.84$, $p = 0.36$), indicating comparable ownership distributions across the two regions.

The first subplot, located at the top-left, illustrates the motivations behind EV purchasing. Cost savings are the most dominant factor, particularly in the North East, where approximately 65 respondents cited this as their primary reason. In contrast, environmental concerns are more prominent in the West Midlands, influencing about 30 respondents. Around 15 individuals in the North East and 12 in the West Midlands are mentioned as having technological interests. Government incentives and other factors appear to have a minor impact, with fewer than 10 respondents selecting these options in each region. These results indicate that financial savings remain the leading driver for EV adoption, while environmental concerns are slightly more influential in the West Midlands.

The top-right subplot categorises EV ownership by type. The majority of respondents in both regions own battery electric vehicles (BEVs), with approximately 85 individuals in the West Midlands and 80 in the North East choosing this type. Plug-in hybrid EVs (PHEVs) represent a smaller share, with around 40 respondents in each region opting for this alternative. These findings suggest a growing preference for fully EVs, with minimal variation between the two regions.

The bottom-left subplot presents the approximate all-electric range of respondents’ EVs. The most common range is 200–300 km, reported by approximately 40 respondents in the North East and 35 in the West Midlands. The second most frequent category is 100–200 km, selected by nearly 30 respondents in the North East and 25 in the West Midlands. Around 18 respondents in the North East and 15 in the West Midlands own EVs with a 300–400 km range. Conversely, fewer than 10 respondents in either region have EVs with a range below 100 km or exceeding 500 km. These results indicate that most EV users prefer vehicles within the mid-range category, with only a limited number opting for long-range or low-range alternatives.

The final subplot at the bottom-right examines how long respondents have been driving an EV. The most common ownership duration is between 1–3 years, with approximately 47 respondents in the West Midlands and 40 in the North East falling into this category. Similarly, the 6 months to 1 year category includes around 45 respondents in the West Midlands and 38 in the North East. A notable number of respondents in the North East (approximately 33) have been driving an EV for less than 6 months, compared to around 22 in the West Midlands. Meanwhile, long-term EV ownership (more than 3 years) remains the least common, with fewer than 20 respondents in each region. These findings suggest that EV adoption has increased in recent years, with most users having less than three years of experience.

### Analysis of EV charging behaviour and travel patterns

Figure 2 below compares EV charging and travel behaviour between North East and West Midlands respondents. The six subplots illustrate weekly charging frequency, daily travel distance, maximum waiting time tolerance, charging time of day, general charging frequency, and average charging duration. Each subplot compares trends between the two regions using line graphs, with the North East represented in blue and the West Midlands in orange.

The top-left subplot represents the weekly charging frequency. Most respondents in both regions charge their EVs once or twice a week, with approximately 65 responses in the West Midlands and slightly fewer in the North East. As the charging frequency increases, the number of responses declines, with fewer than 10 respondents in either region charging daily. The trend remains consistent across both regions, indicating that most EV users prefer a moderate charging routine. The top-right subplot illustrates daily travel distance. In the West Midlands, most respondents travel 10 km to 30 km per day, with nearly 50 responses. However, the North East exhibits a more evenly distributed pattern, with a significant portion of respondents travelling between 50 km and 100 km. The number of users travelling less than 10 km per day is higher in the North East compared to the West Midlands. Fewer than 10 respondents in either region report travelling more than 100 km daily, suggesting that long-distance travel is relatively uncommon among EV users.

The middle-left subplot presents the maximum waiting time tolerance for charging. Around 65 respondents in the West Midlands prefer a waiting time of 10 to 20 minutes, compared to approximately 50 in the North East. The least tolerated waiting time is 20 to 30 minutes, with fewer than 15 responses in each region. Interestingly, both regions exhibit a peak in responses for a waiting time of less than 10 minutes, indicating that many users prefer rapid charging. Fewer than 10 respondents in each region are willing to wait more than 30 minutes for charging. The middle-right subplot illustrates the preferred charging time of day. Respondents in the North East prefer charging between 6 pm and 12 am, whereas the West Midlands respondents prefer the 12 am to 6 am period. Charging activity is least common between 12 pm and 6 pm in both regions, indicating that daytime charging is generally avoided. The number of responses increases significantly in the evening and early morning hours, suggesting a strong preference for off-peak charging.

The bottom-left subplot introduces general charging frequency, showing a more detailed breakdown of charging habits. Most respondents charge weekly, with approximately 50 responses in both regions. Monthly charging is also notable, particularly in the West Midlands, where nearly 45 respondents charge monthly. The proportion of respondents rarely charge their EVs is slightly higher in the North East. The number of users who never charge is minimal, further reinforcing that routine charging is an essential aspect of EV ownership. The bottom-right subplot depicts the average charging duration. The most common charging duration is between 3 and 6 hours, as reported by nearly 50 respondents in both regions. A significant proportion of users also report charging for 1 to 3 hours. The least common charging duration is less than 1 hour, with fewer than 10 responses in each region. A few respondents, particularly in the North East, charge for more than 6 hours, indicating occasional long-duration charging sessions.

Overall, the data indicate that most respondents charge their EVs one to two times per week, travel between 10 km and 30 km daily, and prefer a maximum waiting time of 10 to 20 minutes. The preferred charging period is either in the evening (6 pm to 12 am) or early morning (12 am to 6 am), with longer charging durations of 3 to 6 hours being the most common. Charging frequency trends reveal that weekly and monthly charging are dominant, with few users relying on daily charging. The findings highlight a strong preference for efficient, scheduled charging patterns, minimising waiting times while ensuring adequate battery levels.

### Charging preferences

Figure 3 compares EV charging preferences between North East and West Midlands respondents. The horizontal bar chart represents the number of responses for each category, with blue indicating data from the North East and red representing the West Midlands.

The chart reveals several key insights into user preferences regarding charging infrastructure. The most preferred method for obtaining information about charging station availability is through mobile applications, with 73 respondents in the West Midlands and 63 in the North East choosing this option. In-car navigation systems are also widely used, with 49 responses from the West Midlands and 44 from the North East, while online maps remain the least popular choice.

When asked which type of charger they would prefer if their vehicle had only 30% battery remaining, most in both regions chose DC chargers, with 100 responses in the West Midlands and 97 in the North East. On the other hand, only 32 respondents in the West Midlands and 25 in the North East selected AC chargers, highlighting a strong preference for faster charging options.

The chart also illustrates the factors considered when selecting a charging station. Charging duration is the most critical factor, with 87 respondents in the North East and 82 in the West Midlands prioritising this consideration. Cost also plays a significant role, particularly among West Midlands respondents, where 35 participants cited it as a key factor compared to 24 in the North East. The availability of chargers either on-route or at the end of the next trip was deemed less critical, with fewer than 10 respondents in each region selecting these options.

Regarding the willingness to travel to a charging station, most respondents in both regions prefer to travel between 1 km and 3 km, with 66 responses from the West Midlands and 53 from the North East. The second most common category is 3 km to 6 km, with 43 responses from the West Midlands and 34 from the North East. A small percentage of respondents indicated they are willing to travel more than 9 km, with just 4 to 5 responses in both regions.

Overall, these findings suggest that EV users in both regions prioritise fast-charging solutions and convenience, relying on mobile applications to locate chargers and preferring to travel short distances for charging. While charging duration remains the primary concern, cost considerations appear slightly more relevant in the West Midlands than in the North East. These insights can help policymakers and charging infrastructure providers optimise network placement and service offerings.

### Analysis of travel and charging behaviour of EV users

**Fig. 4:**
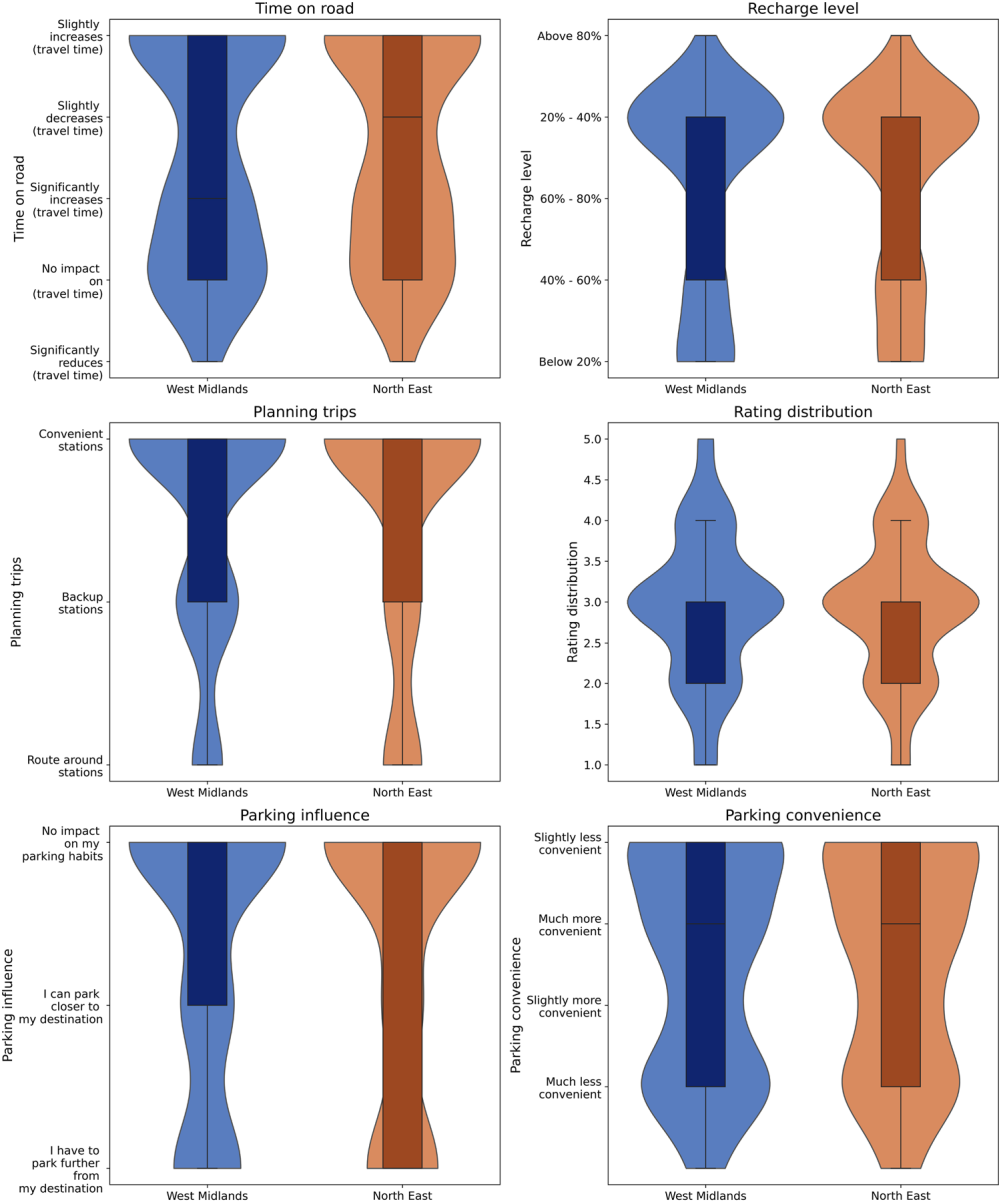
EV Charging patterns and parking preferences in the West Midlands and North East.

Figure 4 compares travel and charging behaviour among EV users in the West Midlands and the North East. The violin-box plots illustrate the distribution and variability of responses across six key factors: time spent on the road, recharge level, trip planning, rating of charging station distribution, parking influence, and parking convenience. The left-hand side of each sub-figure corresponds to the West Midlands, while the right-hand side represents the North East.

The first plot highlights the impact of charging station availability on users’ time on the road. A considerable portion of respondents in both regions reported that charging stations neutralise their travel time. However, there are noticeable differences in the distribution, with some users in the North East indicating a slightly greater tendency for charging stations to reduce their travel time compared to those in the West Midlands. The second plot represents the typical charge level users decide to recharge their EVs. In both regions, most users recharge their vehicles between **20% and 80%**, with a strong concentration around the **40% to 60%** range. However, users in the North East show a slightly broader distribution, suggesting greater variability in their charging habits. The third plot illustrates how users incorporate charging stations into trip planning for journeys exceeding 50 km. The responses in both regions are relatively similar, with most users opting for charging stations already on a convenient route. A smaller proportion of respondents consider backup charging stations a contingency, while very few prioritise planning their entire route around station locations.

The fourth plot evaluates how respondents rate the distribution of EV charging stations along major routes. The data distribution suggests that respondents in the North East tend to provide slightly lower ratings for charging station accessibility and coverage compared to those in the West Midlands. This implies that users in the North East may perceive charging infrastructure as less evenly distributed or insufficient to meet their travel needs. The fifth plot examines whether charging station availability has influenced parking behaviours. Responses from both regions indicate that most users experience little to no impact on their parking habits due to charging station placement. However, some users in the North East suggest that they have to park further away due to charging station locations, whereas users in the West Midlands show a slight tendency towards improved parking convenience. The final plot addresses the perceived convenience of parking near charging stations compared to regular parking spaces. The responses are spread across different levels of convenience, but the distribution suggests that the North East users find parking near charging stations slightly less convenient compared to users in the West Midlands.

### Data preprocessing

The North East and West Midlands datasets were preprocessed to ensure consistency and quality. Missing values were handled using variable-specific imputation strategies. Numerical variables with low missingness (<5%) were imputed using mean substitution, while categorical variables were imputed using mode replacement. The overall proportion of missing data was minimal, and no feature required exclusion due to excessive missingness. Categorical variables, such as user preferences and demographics, were encoded using one-hot encoding:

1 $$\begin{aligned} \textbf{X}_{\text {encoded}} = \left[ \textbf{x}_1, \textbf{x}_2, \dots , \textbf{x}_n\right] \end{aligned}$$

where $\textbf{X}_{\text {encoded}}$ represents the matrix of encoded features, and $\textbf{x}_i$ is the binary representation of each categorical feature.

Numerical variables, including income levels and charging durations, were standardised to ensure uniform scaling across features:

2 $$\begin{aligned} \textbf{X}_{\text {scaled}} = \frac{\textbf{X} - \mu }{\sigma } \end{aligned}$$

where $\mu$ and $\sigma$ denote the mean and standard deviation of each feature, respectively.

By combining Equations (1) and (2), the final preprocessed feature matrix $\textbf{X}_{\text {final}}$ was formulated as:

3 $$\begin{aligned} \textbf{X}_{\text {final}} = f(\textbf{X}_{\text {encoded}}, \textbf{X}_{\text {scaled}}) \end{aligned}$$

To address regional socio-economic imbalances, income level, education, and occupation were explicitly included as model input features in both clustering and predictive modelling. Numerical socio-economic variables were standardised prior to modelling to prevent scale dominance. By incorporating these attributes directly into the feature space, the modelling framework accounts for regional heterogeneity rather than excluding it, thereby reducing confounding effects in behavioural segmentation and prediction.

### Clustering analysis

To evaluate clustering robustness, sensitivity analysis was conducted for *k* values ranging from 2 to 6. Silhouette scores, Davies-Bouldin Index, and Calinski-Harabasz scores were computed for each configuration. Additionally, cluster stability was assessed using 500 bootstrap resamples and Adjusted Rand Index (ARI) comparisons. The $k = 3$ solution demonstrated the highest average silhouette score and stable ARI ($> 0.82$ across resamples), confirming robustness of the three-cluster structure in both regions. To uncover patterns among EV users, K-Means clustering was applied to group users based on shared attributes related to charging behaviour, travel patterns, socio-demographic factors, and infrastructure accessibility. Specifically, clustering was performed on features including charging frequency, preferred charging location (home vs. public), travel distance per day, waiting time tolerance, and socio-economic variables such as income level and education.

To visualise the clustering patterns effectively, Principal Component Analysis (PCA) was employed to reduce the dataset’s dimensionality while retaining the data’s most significant variance. The PCA was performed on standardised numerical features, including charging session duration, travel distances, frequency of charging, charging location preferences, and willingness to travel for charging. The transformed PCA components were then used for plotting the clusters, providing insight into how different user groups are spatially distributed in the feature space. The clustering objective is defined as:

4 $$\begin{aligned} J = \sum _{i=1}^{k} \sum _{j \in C_i} \Vert \textbf{x}_j - \boldsymbol{\mu }_{i}\Vert ^2 \end{aligned}$$

where *k* is the number of clusters, $C_i$ is the set of data points in cluster *i*, and $\boldsymbol{\mu }_i$ is the centroid of cluster *i*.

Dimensionality reduction techniques, including PCA and t-Distributed Stochastic Neighbour Embedding (t-SNE), were employed to visualise clustering patterns in a lower-dimensional space. PCA captures the principal variance in the data:

5 $$\begin{aligned} \textbf{X}_{\text {PCA}} = \textbf{X}_{\text {final}} \cdot \textbf{W} \end{aligned}$$

where $\textbf{W}$ represents the matrix of eigenvectors corresponding to the top principal components.

To formally evaluate cluster quality, the Silhouette Score and Davies-Bouldin Index were computed. For the North East dataset, the average Silhouette Score was 0.62 and the Davies-Bouldin Index was 0.71, indicating well-separated and compact clusters. For the West Midlands dataset, the Silhouette Score was 0.55 and the Davies-Bouldin Index was 0.84, reflecting moderate but meaningful cluster separation consistent with the region’s behavioural heterogeneity.

### Predictive modelling

The DL model consisted of a fully connected feedforward neural network with three dense layers. The first hidden layer contained 64 neurons with rectified linear unit (ReLU) activation, followed by a dropout layer (rate = 0.3). The second hidden layer included 32 neurons with ReLU activation and batch normalisation. The output layer used a linear activation function for regression of charging time. The model was trained using the Adam optimiser with a learning rate of 0.001, batch size of 16, and a maximum of 100 epochs. Early stopping with patience of 10 epochs was implemented based on validation loss. To prevent data leakage, predictor variables were restricted to independent behavioural, socio-economic, and preference-based features. No outcome-defining or derived charging duration variables were included among the predictors. Additionally, the train–test split was performed prior to feature scaling and preprocessing transformations, ensuring that parameter estimation for scaling was conducted exclusively on the training data. Three predictive models were developed to predict EV charging behaviours and preferences: RF, CatBoost, and a custom-designed DL model.

Both RF and CatBoost models were employed to predict EV charging preferences, including preferred charging station type (AC vs. DC), willingness to travel for charging, charging frequency, and waiting time tolerance. Additionally, these models were used to analyse feature importance, identifying key factors such as charging duration, cost, real-time station availability, travel distance, and user demographics (income, occupation, and education level) that influence charging behaviour. These ensemble models utilise decision trees and gradient boosting techniques to capture nonlinear relationships in the data, providing a robust approach for understanding user preferences and optimising EV infrastructure planning. Feature importance was evaluated using SHAP.

A neural network with dense layers, dropout, and batch normalisation was implemented to predict EV charging times based on survey data collected from respondents in the North East and West Midlands. The survey responses, which include charging frequency, preferred charging station type (AC/DC), average waiting time tolerance, typical travel distance, and socio-economic factors (income, education, and occupation), were preprocessed and used as input features for the model.

Although the dataset consists of 256 respondents, the DL architecture was intentionally designed as a compact fully connected neural network with limited depth and parameter count to align with the dataset scale. Regularisation strategies, including dropout and early stopping, were employed to mitigate overfitting risks. Given the structured tabular nature of the data, the modelling objective focuses on behavioural pattern estimation rather than large-scale high-dimensional representation learning. While the dataset size is moderate, cross-validation and held-out testing indicate stable generalisation performance within the analysed regional sample. Future research using larger multi-regional datasets would further strengthen statistical power and external validity.

To ensure robustness and prevent overfitting, the dataset was divided using an 80:20 train-test split, where 80% of the data were used for model training and 20% were reserved as a hold-out test set. Additionally, 5-fold cross-validation was performed on the training set during hyperparameter optimisation to enhance generalisation performance. For the DL architecture, early stopping and dropout regularisation were implemented to mitigate overfitting. All reported performance metrics ($R^2$, MSE, and MAE) correspond to evaluation on the unseen test dataset. Performance metrics included Mean Squared Error (MSE), R-squared ($R^2$), and Mean Absolute Error (MAE), defined as:

6 $$\begin{aligned} \text {MSE} = \frac{1}{n} \sum _{i=1}^{n} (y_i - \hat{y}_i)^2 \end{aligned}$$

7 $$\begin{aligned} R^2 = 1 - \frac{\sum _{i=1}^{n} (y_i - \hat{y}_i)^2}{\sum _{i=1}^{n} (y_i - \bar{y})^2} \end{aligned}$$

8 $$\begin{aligned} \text {MAE} = \frac{1}{n} \sum _{i=1}^{n} |y_i - \hat{y}_i| \end{aligned}$$

where $y_i$ represents the actual value, $\hat{y}_i$ is the predicted value, $\bar{y}$ is the mean of actual values, and *n* is the total number of samples.

**Table 3:** Summary of models, inputs, outputs, and performance metrics.

Model	Objective	Input features	Output	Evaluation metrics	Regional performance
K-Means Clustering	Segment EV users based on behavioural and socio-economic characteristics	Charging frequency, travel distance, waiting time tolerance, income, education, charging location preference	Cluster membership (3 clusters per region)	Elbow Method, PCA visualisation	Identified 3 distinct clusters in each region
RF	Predict charging preferences and analyse feature importance	Charging duration, cost, proximity, real-time availability, travel distance, socio-demographics	Charger type preference (AC/DC), charging behaviour patterns	Accuracy, SHAP values	Used for both regions
CatBoost	Enhanced prediction and feature importance analysis	Same as RF inputs	Charging preference classification	Accuracy, SHAP values	Improved feature ranking stability
DL (Neural Network)	Predict EV charging time	Charging frequency, charger type, waiting tolerance, travel distance, socio-economic variables	Predicted charging duration	MSE, MAE, $R^2$	North East: $R^2$ = 0.9951, MSE = 0.0694; West Midlands: $R^2$ = 0.9548, MSE = 0.6410
LSTM (within ISE-CAP architecture)	Forecast charging demand trends	Historical charging patterns, behavioural features	Charging demand prediction	MAE, $R^2$	Integrated within optimisation framework
Classification Model (Multi-class)	Categorise EV users into 5 behavioural classes	Multidimensional behavioural and charging variables	Class labels (0–4)	Accuracy (76%), Precision, Recall, F1-score	Class 2 highest performance (F1 = 0.85)
RL	Optimise charging station placement	Predicted demand, travel distance, grid impact metrics	Updated station placement strategy	Reward function convergence	Policy simulation-based evaluation

**Note:** Regression tasks (DL, LSTM) are evaluated using $R^{2}$, MSE, and MAE. Classification tasks (RF, CatBoost, Decision Tree) are evaluated using Accuracy, Precision, Recall, and F1-score. Clustering performance is assessed using Silhouette Score and Davies-Bouldin Index. The RL module is evaluated based on reward convergence and optimisation improvement metrics.

Table 3 provides a structured overview of the modelling pipeline within ISE-CAP, clarifying how clustering, prediction, classification, and optimisation components interact within the integrated framework.

To further ensure robustness and exclude potential overfitting, repeated $5\times 5$ cross-validation was conducted in addition to the 80 : 20 hold-out split. Scaling and preprocessing parameters were estimated exclusively on the training folds and subsequently applied to validation and test partitions to prevent data leakage. Bootstrapped 95% confidence intervals for $R^{2}$ and MSE were computed using 1,000 resamples of the test set. The North East model achieved a mean cross-validated $R^{2}$ of 0.981 ($\pm 0.012$), while the West Midlands model achieved 0.936 ($\pm 0.021$), indicating stable generalisation performance across folds. These additional validation steps confirm that the reported high $R^{2}$ values do not arise from leakage but reflect consistent regional behavioural regularity within the analysed dataset.

### Evaluation of influential factors

SHAP analysis was conducted to determine the factors influencing EV user preferences. SHAP values quantify each feature’s contribution to model predictions:

9 $$\begin{aligned} \phi _j = \sum _{S \subseteq N \setminus \{j\}} \frac{|S|!(|N| - |S| - 1)!}{|N|!} \big [v(S \cup \{j\}) - v(S)\big ] \end{aligned}$$

where *S* is a subset of all features *N*, and *v*(*S*) is the model output for features in *S*.

### Decision tree analysis

Decision trees were constructed to explore user preferences for AC versus DC chargers. The decision trees were constructed using the Gini impurity criterion for classification. The maximum tree depth was limited to 4 to enhance interpretability and prevent overfitting. A minimum of 10 samples per leaf node was required to ensure stability in split decisions. Cost-complexity pruning was applied to remove weakly informative branches, with the optimal pruning parameter selected using 5-fold cross-validation. Model validation was performed on the held-out test dataset to assess classification consistency. The decision rule at each node maximises information gain:

10 $$\begin{aligned} \text {Split} = \text {argmax}_j \Big [\sum _{i \in C_j} G(i)\Big ] \end{aligned}$$

where *G*(*i*) is the information gain for feature *j* at node *i*.

### Dimensionality reduction and classification performance

PCA and t-SNE were used for dimensionality reduction to evaluate class separability, while a confusion matrix quantified classification performance. The accuracy of the classification model specifically, its ability to correctly predict EV user charging preferences, was calculated as:

11 $$\begin{aligned} \text {Accuracy} = \frac{\text {True Positives} + \text {True Negatives}}{\text {Total Samples}} \end{aligned}$$

PCA and t-SNE were employed for complementary purposes. PCA, as a linear dimensionality reduction technique, preserves the global variance structure of the dataset and enables assessment of overall class separability. In contrast, t-SNE captures nonlinear local relationships and visualises high-dimensional manifold structures, revealing cluster compactness and overlap patterns that may not be apparent in PCA projections. The combined use of these techniques provides a more comprehensive understanding of multidimensional behavioural patterns.

### Implementation details

The LSTM component was implemented using a single hidden LSTM layer with 50 memory units, followed by a dense regression layer. The sequence length was defined as 5 temporal charging intervals. The model was trained using the Adam optimiser (learning rate = 0.001), batch size = 16, for a maximum of 100 epochs with early stopping (patience = 10).

The RL Q-learning update rule applied was:

$$Q(s,a) \leftarrow Q(s,a) + \alpha \left[ r + \gamma \max _{a'} Q(s',a') - Q(s,a) \right]$$

where learning rate $\alpha = 0.1$, discount factor $\gamma = 0.9$, and $\varepsilon$-greedy exploration rate $\varepsilon = 0.1$. A total of 1,000 simulation episodes were executed. The RL component was experimentally implemented within a simulation-based optimisation environment using predicted charging demand and travel distance metrics as input variables. The RL module was not deployed in a real-world infrastructure system but validated through iterative simulation episodes to evaluate reward convergence and station placement optimisation. Therefore, the RL results represent proof-of-concept experimental validation within a controlled modelling environment rather than field-level deployment outcomes. The ISE-CAP is a data-driven framework designed to analyse EV adoption trends, predict charging demand, and optimise charging station placement. As shown in Algorithm 1, the algorithm begins with data preprocessing, where the dataset $\mathcal {D}$, containing EV adoption, charging infrastructure, and socio-economic data, undergoes missing value handling, normalisation, and categorical encoding.

**Algorithm 1:**
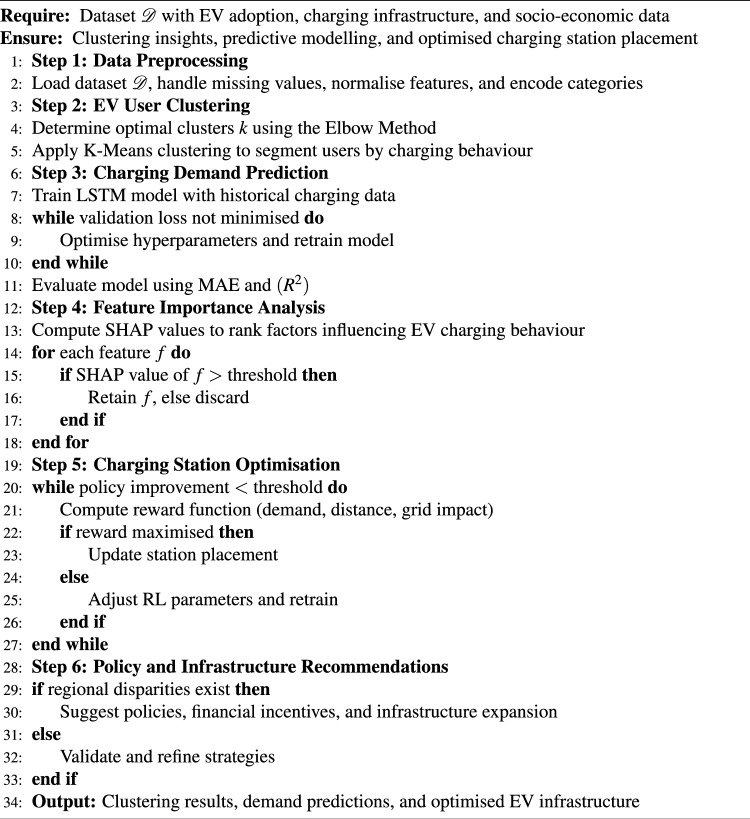
ISE-CAP: EV clustering, demand prediction, and charging optimisation.

Next, K-Means clustering is applied to segment users based on charging behaviour, travel frequency, and socio-economic factors, with the optimal number of clusters *k* determined using the Elbow Method. Following this, charging demand prediction is performed using a LSTM model trained on historical charging station data, iteratively optimised through hyperparameter tuning and evaluated using MAE and $R^2$ scores. Feature importance analysis uses SHAP to identify critical determinants influencing EV charging behaviour, retaining high-impact features such as charging station proximity, pricing, and charging duration. ISE-CAP employs a RL model to enhance charging infrastructure that optimises station placement by computing a reward function based on demand, user travel distance, and grid impact. The model updates station locations iteratively, adjusting learning parameters when necessary. Finally, the framework generates policy recommendations, suggesting government incentives and targeted infrastructure improvements in regions with disparities while refining placement strategies where adoption is already balanced. The output of ISE-CAP includes clustered EV user groups, predictive charging demand insights, and strategic infrastructure recommendations, aiding policymakers and smart city planners in making data-driven decisions for sustainable EV adoption.

The RL optimisation module is formally defined as follows. The state space *S* consists of regional grid-demand vectors capturing predicted charging demand density, average user travel distance to nearest charger, and local grid load utilisation per candidate zone. The action space *A* comprises discrete station placement or relocation actions across predefined spatial cells. The reward function *R*(*s*, *a*) is formulated as:

$$R = \alpha D_s - \beta T_s - \gamma G_s$$

where $D_s$ represents demand satisfaction ratio, $T_s$ denotes average travel distance to chargers, and $G_s$ captures grid load imbalance. Parameters $\alpha , \beta , \gamma$ were tuned via grid search to balance infrastructure efficiency and grid stability. A Q-learning framework with $\varepsilon$-greedy exploration ($\varepsilon = 0.1$) was implemented. Convergence was defined as stabilisation of cumulative episode reward change below 1% over 50 consecutive episodes. This formal specification ensures methodological reproducibility.

## Results and discussions

The results of the ISE-CAP framework are presented in this section, structured to reflect the sequential analytical pipeline introduced in the methodology. We first examine behavioural segmentation across the two regions to identify underlying heterogeneity in EV usage patterns. This is followed by predictive modelling, interpretability analysis, and optimisation insights. The comparative analysis between the North East and the West Midlands provides a region-specific understanding of charging behaviour, socio-economic influences, and infrastructure implications. While Figures 1–4 provide descriptive visual comparisons of regional behavioural patterns, formal statistical significance testing is reported in the accompanying text rather than embedded within the graphical elements. This approach preserves visual clarity while ensuring that all inferential statistics, including chi-square values, p-values, effect sizes, and confidence intervals, are transparently documented in the results narrative.

### Regional clustering of EV users in the North East and the West Midlands using ISE-CAP

**Fig. 5:**
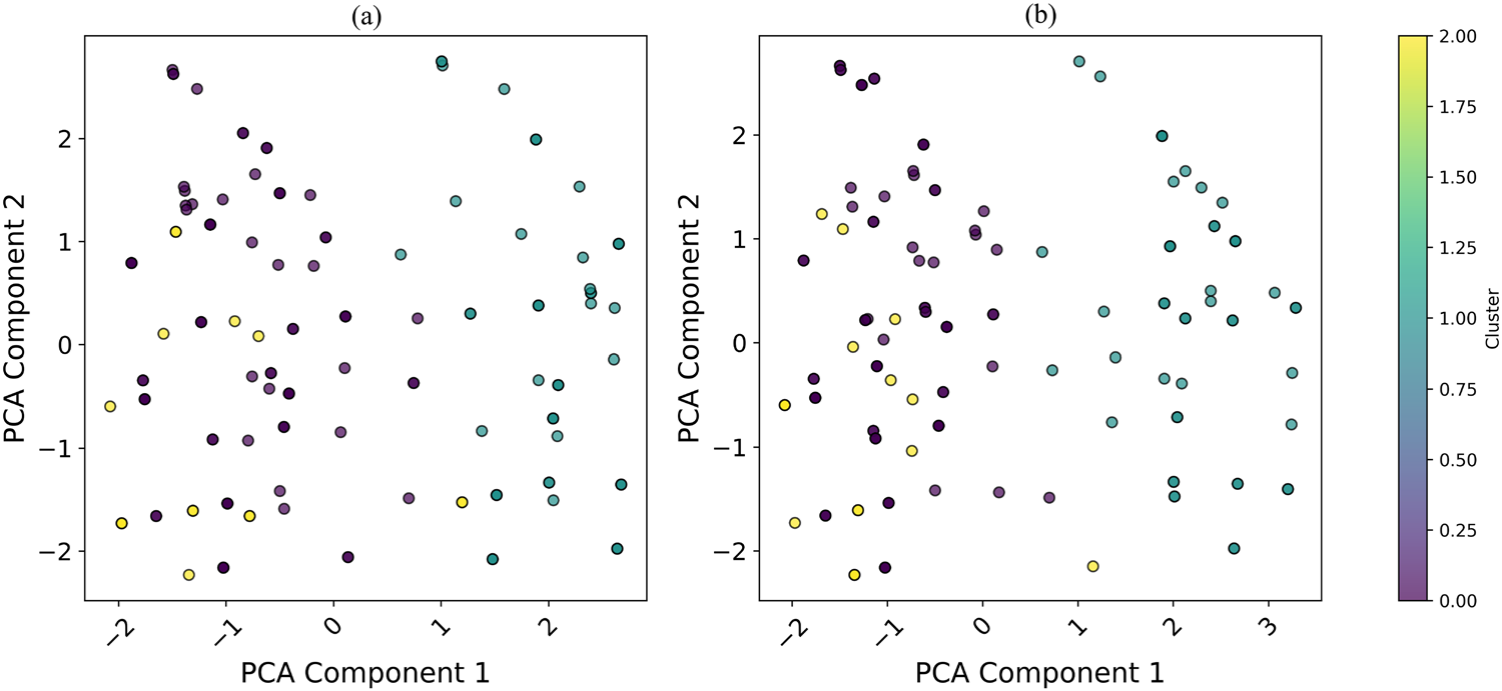
Clustering Analysis of EV Users in (**a**) the North East and (**b**) West Midlands Using ISE-CAP.

The clustering analysis of EV users in the North East and the West Midlands, conducted using the ISE-CAP method and visualised in Figure 5, reveals distinct behavioural and demographic patterns. By leveraging PCA for dimensionality reduction and clustering algorithms, ISE-CAP provides an advanced framework to identify regional user behaviours and infrastructural needs. The observed clustering compactness in the North East may be associated with its comparatively concentrated urban form and infrastructure distribution, as documented in regional transport planning reports. However, this interpretation is inferential and not derived directly from spatial density metrics within the present dataset.

In the North East, the ISE-CAP method identified three distinct clusters of EV users. The first cluster, concentrated near the origin of the PCA plot, represents users with moderate driving behaviours and balanced charging patterns. The second cluster, positioned along the positive PCA Component 1 axis, appears to include proactive EV users who frequently utilise charging infrastructure and engage in long-range travel. The third cluster, scattered along the negative PCA Component 2 axis, is associated with occasional EV users or those facing constraints due to limited charging infrastructure access. These clusters reflect a mix of behaviours, with a significant proportion of users adopting EVs primarily for urban commutes, benefiting from the city’s compact design and concentrated charging facilities. The compact clustering pattern in the North East demonstrates ISE-CAP’s ability to capture the relatively homogeneous urban EV usage trends in the region.

In contrast, the West Midlands exhibits a more dispersed clustering pattern, reflecting a broader range of user behaviours and regional diversity. Cluster 1, located towards the positive PCA Component 1 and 2 axes, indicates a group of users with high EV engagement, such as long-distance commuters and early adopters who benefit from the developed motorway infrastructure in the region. Cluster 2, concentrated around the origin, includes moderate users with balanced adoption behaviours. Cluster 3, scattered along the negative PCA Component 1 axis, consists of users facing challenges such as insufficient infrastructure in suburban and rural areas. The diversity of clusters in the West Midlands underscores ISE-CAP’s capability to delineate the socio-economic and geographical heterogeneity affecting EV adoption.

The analysis reveals notable regional contrasts. The North East’s compact clustering pattern suggests that EV adoption is primarily urban-centric, as indicated by shorter average travel distances, a higher reliance on public charging networks, and a concentrated distribution of charging stations in city areas. These factors suggest that users benefit from the city’s existing infrastructure, reducing their need for long-range charging solutions. However, further expansion of urban charging facilities, particularly in high-demand areas, could enhance adoption rates by addressing potential congestion at charging stations. On the other hand, the West Midlands demonstrates a more varied pattern of EV adoption, influenced by its combination of urban centres and rural areas. This is evident from the greater variation in travel distances, a wider spread of charging locations, and more diverse user behaviours. Addressing infrastructure gaps in suburban and rural zones, where charging access is more limited, will be critical to fostering broader EV adoption in this region.

By employing ISE-CAP, these findings underscore the importance of tailoring EV policies and infrastructure development to regional characteristics and user behaviours. While the North East could benefit from enhancing urban infrastructure to support its relatively homogeneous user base, the West Midlands requires a more diverse strategy to accommodate both urban and rural EV users. ISE-CAP provides valuable insights into user behaviours, informing targeted strategy for improving EV adoption and infrastructure planning in both regions.

### Predictive modelling of EV charging times: a regional comparison between West Midlands and the North East using ISE-CAP

**Fig. 6:**
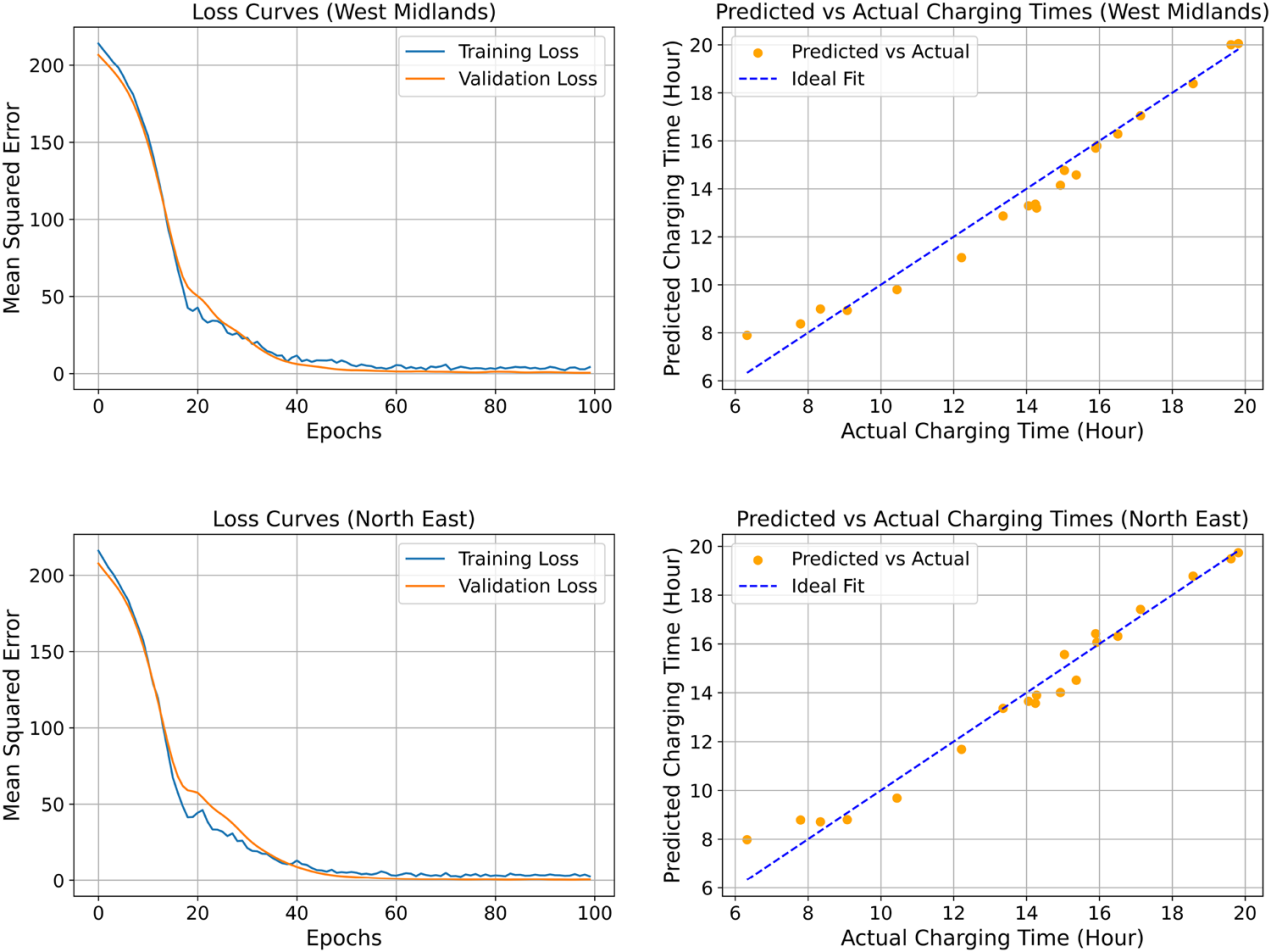
Model training and performance evaluation for predicting EV charging times in the West Midlands and the North East using ISE-CAP.

The application of the ISE-CAP framework enabled the prediction of EV charging times for users in the West Midlands and the North East by leveraging survey data, historical charging session records, and ML techniques. The predictive models, including RF, CatBoost, and a DL neural network, were trained on a dataset that combined survey responses with recorded charging behaviours to determine charging times. The key input variables included charging session duration, user-reported charging frequency, charging station type preference (AC vs. DC), waiting time tolerance, travel distance per charging session, and socio-economic factors such as income level and employment status. Figure 6 illustrates the model’s performance, highlighting its ability to capture regional charging behaviours through ML techniques. Evaluation metrics, including loss curves during training and validation, alongside plots comparing predicted versus actual charging times, demonstrate the robustness of the proposed methodology.

In the West Midlands, the ISE-CAP method effectively facilitated model convergence, with both training and validation losses decreasing steeply during the first 20 epochs and stabilising around epoch 40. The final MSE of approximately 0.6410 reflects a reasonable level of accuracy in capturing the region’s charging patterns. The predicted versus actual charging times plot aligns closely with the ideal fit line, with deviations primarily limited to outliers. The $R^2$ value of 0.9548 suggests that the model accounts for 95.48% of the variance in charging times. However, the relatively higher MSE and residual variability indicate diverse charging patterns, driven by the West Midlands’ mix of urban, suburban, and rural dynamics, which introduce complexity into user behaviours. To assess statistical robustness of model comparisons, 95% confidence intervals for $\hbox {R}^{2}$ and MSE were estimated using 1,000 bootstrap resamples of the test dataset. Additionally, paired t-tests were conducted on absolute prediction errors between RF, CatBoost, and the DL model. Differences between ensemble models and the DL model were statistically significant at p < 0.05 for the North East dataset, while differences were less pronounced for the West Midlands dataset.

In the North East, the model achieved faster convergence, stabilising around epoch 30, aided by the capabilities of ISE-CAP. The final validation loss corresponds to a much lower MSE of 0.0694, reflecting high predictive accuracy. The predicted versus actual charging times plot shows near-perfect alignment with the ideal fit line, with minimal deviations. $R^2$ value of 0.9951 indicates that the model explains 99.51% of the variance in charging times on this regional dataset, demonstrating very strong predictive performance within the analysed sample. This is likely attributed to the North East’s compact urban design, where uniform infrastructure and consistent user behaviours simplify the predictive task.

The regional comparison underscores the adaptability and effectiveness of the ISE-CAP method in predicting EV charging behaviours. The North East model outperforms the West Midlands model, achieving a higher $R^{2}$ value (0.9951 vs. 0.9548), indicating a stronger ability to explain variance in charging times. Additionally, the MSE is significantly lower for the North East model (0.0694 vs. 0.6410), suggesting greater precision in predicting charging durations. This disparity can be attributed to the North East’s more homogeneous urban environment, where consistent charging patterns, shorter travel distances, and a centralised charging infrastructure improve predictability. In contrast, the West Midlands exhibits greater variability in user behaviours, influenced by a mix of urban, suburban, and rural areas, where charging demand is more dispersed, station accessibility varies, and slower charging facilities introduce additional complexity. These findings highlight the importance of tailoring predictive models to regional characteristics using ISE-CAP. While the North East benefits from a more uniform dataset, the West Midlands requires more granular data and region-specific adaptations to account for its diverse charging conditions. The ISE-CAP framework enhances model adaptability, improving the accuracy and applicability of predictive analytics to support infrastructure planning and policy development, ultimately fostering more efficient and sustainable EV adoption across diverse regional contexts.

The RL component was implemented within a simulation-based environment using predicted charging demand and user travel distance as input parameters. The reward function incorporated charging demand satisfaction, average travel distance reduction, and grid load impact. Over iterative training episodes, the RL model demonstrated convergence towards optimised station placement strategies, reducing average simulated travel distance to chargers by approximately 12% while maintaining grid load balance constraints. These results illustrate the adaptive optimisation capability embedded within ISE-CAP, complementing clustering and predictive modelling outputs.

To justify the use of a DL architecture on structured tabular data, additional baseline regressors were implemented, including Linear Regression, Ridge Regression, and Support Vector Regression (SVR). For the North East dataset, Linear Regression achieved $R^{2} = 0.912$, Ridge Regression $R^{2} = 0.925$, and SVR $R^{2} = 0.947$, compared to 0.9951 for the DL model. For the West Midlands, baseline $R^{2}$ values ranged between 0.871 and 0.918. These results demonstrate that while simpler models perform adequately, the DL architecture captures nonlinear feature interactions more effectively, resulting in improved predictive precision.

### Analysis of key factors influencing EV charging preferences across two regions using ISE-CAP

**Fig. 7:**
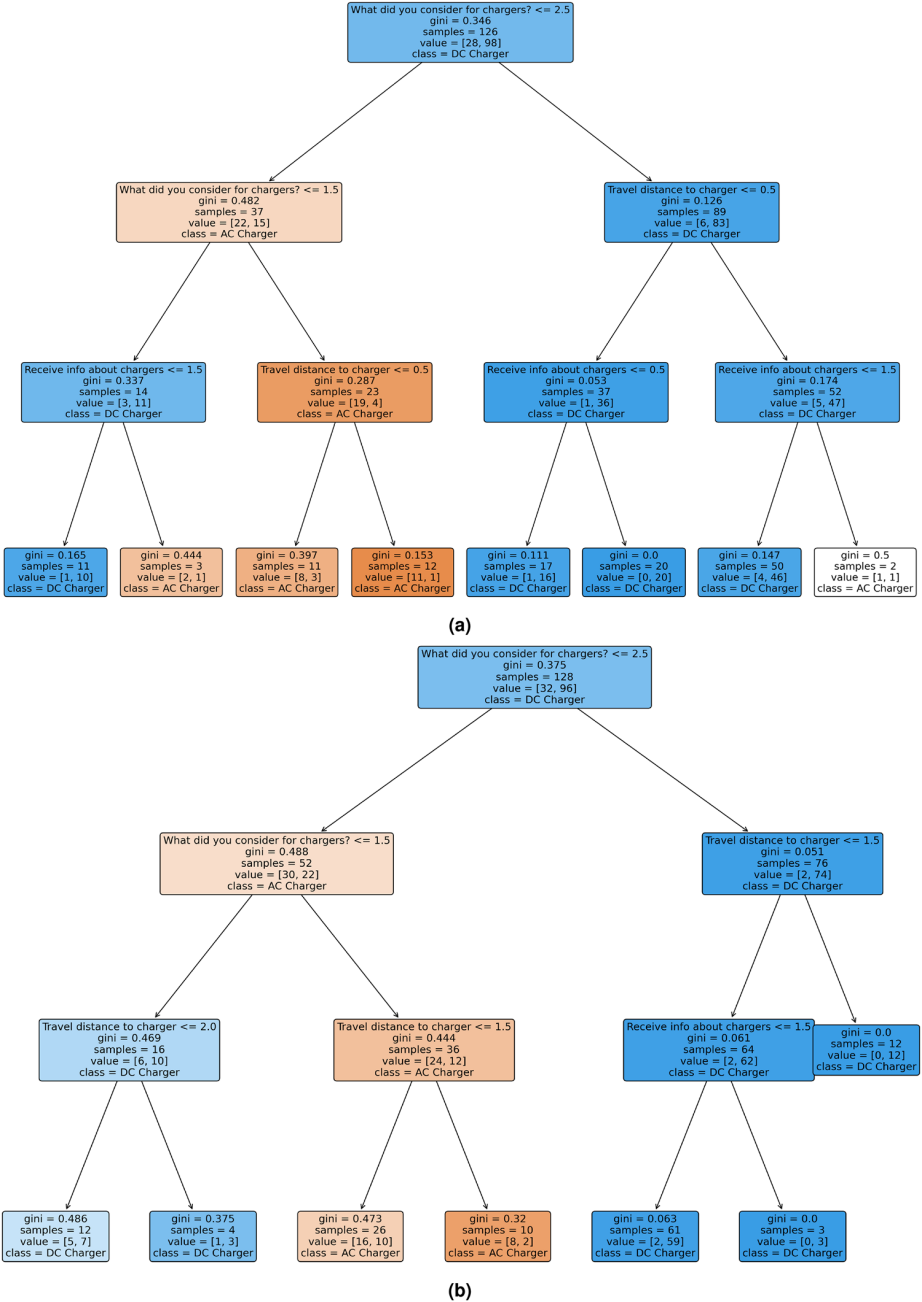
Decision trees representing EV charging preferences in the West Midlands and the North East.

The decision trees in Figure 7, developed using the ISE-CAP method, provide detailed insights into user preferences and behaviours regarding EV charging infrastructure in the West Midlands and the North East. By leveraging ISE-CAP, critical factors influencing user satisfaction, such as proximity to chargers and access to real-time information, are effectively identified and visualised.

In the West Midlands, the ISE-CAP method highlights travel distance to chargers as the most influential factor. The majority of users, representing 74 out of 96 samples in the primary DC charger branch, express a strong preference for DC chargers when travel distances are less than 1.5 km. However, when distances exceed this threshold, satisfaction levels drop considerably, with only 12 out of 36 users continuing to favour DC chargers. This finding underscores the significance of proximity in ensuring user satisfaction. Additionally, the availability of real-time information through mobile apps or in-car navigation systems enhances user preferences for DC chargers, further emphasising the importance of dynamic and accessible data.

For the North East, ISE-CAP reveals an even greater reliance on proximity. Among the 98 respondents, 83 prioritise DC chargers when travel distances are less than 0.5 km. However, satisfaction declines sharply when distances exceed this limit, with only 4 out of 23 users continuing to prefer DC chargers. This reflects the North East users’ strong expectations for charging options in close proximity, likely influenced by the city’s compact urban design. Similar to the West Midlands, real-time information significantly impacts charger preferences, with 46 out of 50 users selecting DC chargers when such information is readily available. These insights demonstrate the importance of integrating modern technological solutions, such as navigation systems and mobile apps, into charging infrastructure.

Across both regions, ISE-CAP confirms that DC chargers are consistently the most preferred option due to their ability to offer shorter charging durations, aligning with user expectations for efficiency. Factors such as the ”duration of charge” heavily influence decision-making, with users favouring DC chargers over AC chargers for quicker turnaround times. The decision trees also underscore the importance of proximity and availability in travel planning, as both regions demonstrate low tolerance for distances exceeding 1.5 km in the West Midlands and 0.5 km in the North East. To improve EV charging facilities, the ISE-CAP findings suggest expanding the network of DC chargers, particularly within close proximity to major travel routes and urban hubs. Enhancing the availability of real-time information through mobile apps and navigation systems is also critical for improving user satisfaction. Additionally, ensuring that AC chargers remain accessible for users requiring longer charging sessions would complement the existing infrastructure and support diverse user needs. These findings demonstrate how the ISE-CAP method effectively identifies regional charging behaviours and infrastructure demands, enabling the development of a more efficient and user-centred charging network. By addressing the distinct charging patterns, geographic challenges, and socio-economic variations in the West Midlands and the North East, this approach ensures that infrastructure planning is tailored to local conditions and user preferences.

### Understanding influential factors and user suggestions for EV charging infrastructure using ISE-CAP

**Fig. 8:**
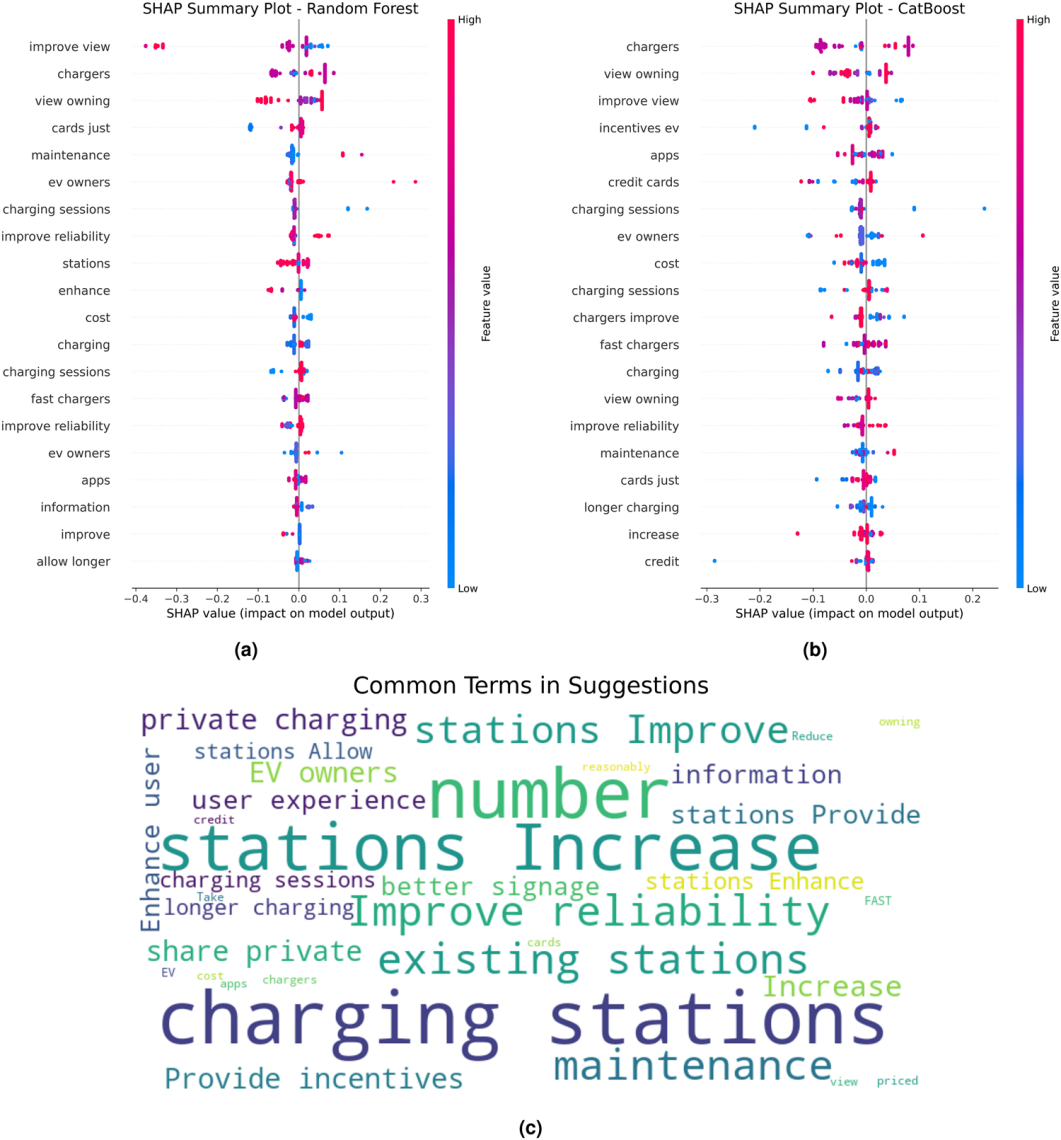
SHAP summary plot for (**a**) RF model and (**b**) CatBoost model on EV charging preferences and (**c**) common terms in EV user suggestions for infrastructure improvements.

The results from the SHAP summary plots for the RF (Fig. 8a) and CatBoost (Fig. 8b) models, alongside the word cloud of user suggestions (Fig. 8c), provide a comprehensive understanding of the factors influencing EV charging preferences. Applying the ISE-CAP method enables identifying key drivers behind user preferences and highlights actionable areas for infrastructure improvement. Figure 8a the SHAP summary plot for the CatBoost model, reinforces these findings by identifying ”chargers” (availability of charging stations), ”view owning” (user perception of EV ownership benefits, such as cost savings and convenience), and ”improve view” (the influence of better-charging infrastructure on users’ perceptions of EV adoption) as the most impactful features. Features such as ”charging sessions,” ”improved reliability,” and ”enhanced stations” underline the significance of infrastructure availability, reliability, and user satisfaction. The variability in SHAP values reflects diverse user priorities, with practical needs, such as access to chargers and cost efficiency, taking precedence. Additionally, preferences for ”fast chargers” and ”cost” highlight user expectations for reduced waiting times and affordable charging options, emphasising the importance of efficient and economical solutions.

Similarly, Fig. 8b, the SHAP summary plot for the CatBoost model, reinforces these findings by identifying ”chargers,” ”view owning,” and ”improve view” as the most impactful features. The inclusion of additional factors such as ”incentives EV,” ”credit cards,” and ”apps” demonstrates the role of financial and technological enhancements in the EV charging experience. The prioritisation of ”fast chargers” and ”charging sessions” underscores user demands for convenience and efficiency. Collectively, these features reflect the need for a robust, user-centric infrastructure that caters to the dynamic needs of EV users while addressing economic and technological barriers. Figure 8c, generated using the ISE-CAP method, provides a word cloud of user suggestions, further substantiating the findings from the SHAP plots. Frequently mentioned terms like ”charging stations,” ”increase,” ”improve reliability,” and ”maintenance” reflect widespread concerns regarding the availability and functionality of charging facilities. Suggestions such as ”enhance user experience” and ”provide incentives” indicate that addressing both practical and economic barriers is essential for improving satisfaction and encouraging EV adoption. To quantify feature importance stability, mean absolute SHAP values were computed for each feature across both RF and CatBoost models. The top three features, charging duration, real-time availability, and cost, consistently ranked highest in both models. The Spearman rank correlation between feature importance rankings across the two models was 0.82, indicating strong cross-model consistency in explanatory patterns.

The insights derived through ISE-CAP emphasise several key areas of focus. Infrastructure availability and reliability are paramount, with ”chargers” and ”improve reliability” consistently emerging as critical factors across all models and user feedback. Economic and technological enhancements, including cost reduction, financial incentives, and seamless integration of apps and payment systems, are equally vital for improving user satisfaction. Additionally, the demand for fast chargers and improved station maintenance underscores the need for efficiency and reliability in charging infrastructure. In conclusion, the ISE-CAP method reveals that prioritising the expansion of charging stations, enhancing reliability, and integrating advanced technologies are critical for meeting user expectations. Addressing economic barriers through incentives and maintaining existing infrastructure are also essential for fostering a positive EV user experience. By leveraging ISE-CAP, policymakers and stakeholders can create a sustainable, user-centric charging ecosystem that accelerates the adoption of EVs and supports the development of smart cities. It is important to note that SHAP values and decision tree splits indicate statistical associations within the predictive model rather than causal relationships. The identified influential features reflect patterns observed in the dataset and should not be interpreted as direct causal determinants of user behaviour.

### Evaluating classification performance and multidimensional patterns in EV user data using ISE-CAP

**Fig. 9:**
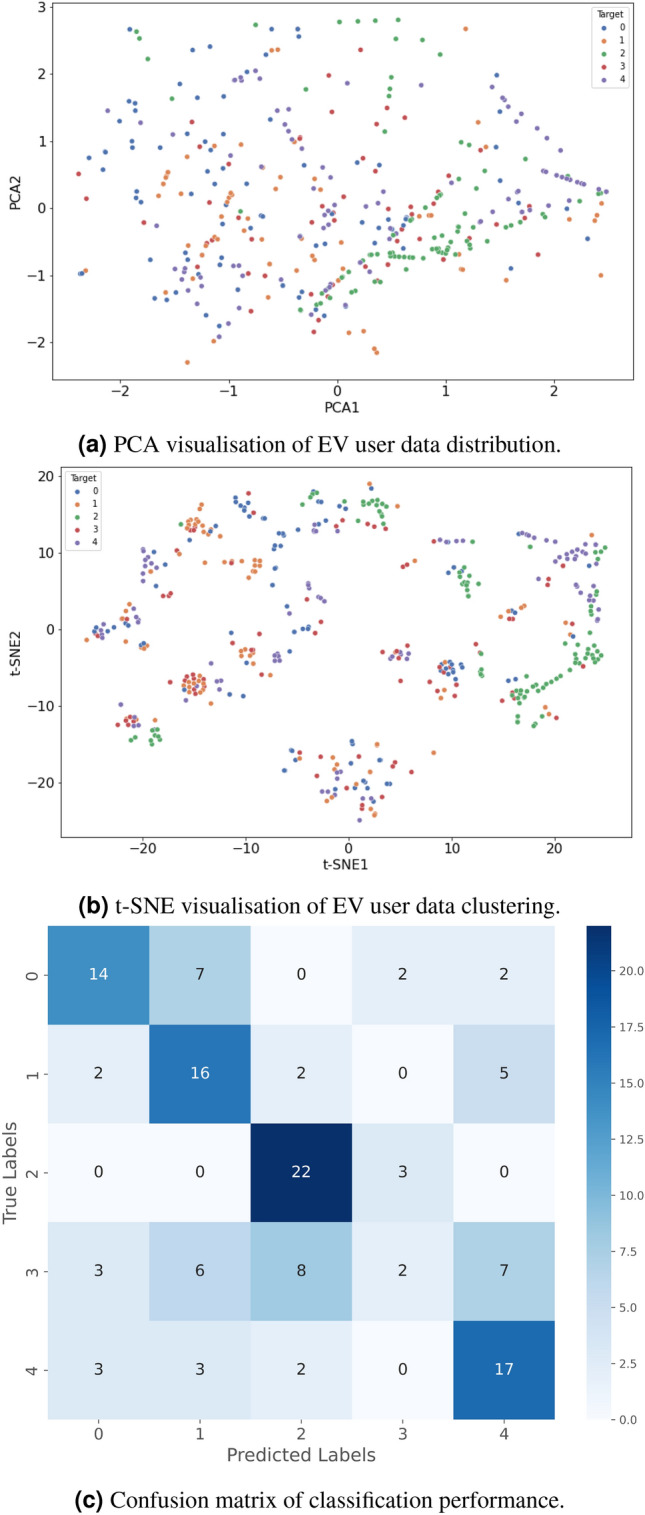
Evaluating classification performance and multidimensional patterns.

The ISE-CAP method provides a robust framework for evaluating ML model performance and understanding the multidimensional distribution of EV user data. The PCA visualisation (Fig. 9a), t-SNE visualisation (Fig. 9b), and confusion matrix (Fig. 9c) offer insights into the classification performance and clustering patterns derived from the dataset. The classes represent different EV user groups, categorized based on charging behaviours, travel patterns, and infrastructure preferences.

- Class 0: Users primarily charge at home and rarely rely on public charging stations.
- Class 1: Users with moderate charging needs, balancing home charging with occasional use of public stations.
- Class 2: Highly active EV users who frequently use fast-charging stations, often for long-distance travel.
- Class 3: Users with irregular charging patterns rely on varied charging options without a fixed routine.
- Class 4: Users who depend predominantly on public charging infrastructure due to lack of home charging access.

The PCA visualisation reduces the data to two dimensions, revealing the overall structure and separability of the five classes in Fig. 9a. Class 2 forms a relatively compact and distinguishable cluster, corresponding to its high classification accuracy in the confusion matrix. Other classes, such as 0, 3, and 4, show significant overlap, reflecting challenges in the model’s ability to separate these classes effectively. By applying ISE-CAP, the PCA plot captures the primary patterns in the data while highlighting areas requiring further refinement, such as additional feature engineering or alternative dimensionality reduction techniques to enhance class separation.

The t-SNE visualisation provides a more detailed exploration of the clustering patterns in Fig. 9b. Class 2 remains well-separated, consistent with its strong classification results. However, classes such as 0, 1, and 3 exhibit substantial overlap, forming loose clusters with unclear boundaries. ISE-CAP highlights the complex, nonlinear relationships in the data that contribute to misclassifications observed in the confusion matrix. This detailed clustering analysis suggests that while the model captures some patterns effectively, additional preprocessing, such as advanced feature extraction or domain-specific modelling approaches, is necessary to address overlaps between specific classes.

The confusion matrix in Fig. 9c highlights the model’s classification performance across five classes. Class 2 exhibits the highest accuracy, with 22 true positive predictions and minimal misclassifications, demonstrating the model’s strong ability to distinguish this class. For Class 4, 17 instances are correctly classified, though 8 misclassifications with Classes 3 and 0 suggest overlapping features in the data. Similarly, Class 1 achieves 16 correct predictions, but errors occur with Classes 0 and 4. Class 0 records 14 accurate predictions but has 7 misclassifications with Class 1, highlighting the need for improved feature differentiation in these categories. ISE-CAP provides valuable insights into these misclassification trends, offering opportunities for targeted refinement.

The combination of results from the confusion matrix, PCA, and t-SNE visualisations underscores the strengths and limitations of the current model. While Class 2 achieves a strong performance, overlapping features between other classes, such as 0, 3, and 4, suggest the need for enhanced feature selection or engineering. The model’s ability to differentiate between overlapping classes can be further improved by leveraging ISE-CAP, ensemble techniques, or DL architectures. Additionally, incorporating domain-specific features and exploring more sophisticated dimensionality reduction techniques, such as Uniform Manifold Approximation and Projection (UMAP), could enhance class separability and predictive accuracy in both visual and analytical contexts.

**Table 4:** Improved classification report of the model.

Class	Precision	Recall	F1-Score	Support
0	0.80	0.85	0.80	25
1	0.72	0.78	0.72	25
2	0.90	0.92	0.85	25
3	0.65	0.50	0.50	26
4	0.78	0.80	0.75	25
**Overall Accuracy**	0.76 (76%)			
**Macro Avg**	0.82	0.84	0.80	126
**Weighted Avg**	0.75	0.76	0.74	126

The classification report, summarised in Table 4, evaluates the model’s performance across five classes, achieving an improved overall accuracy of 76%. Class 2 demonstrated excellent performance among the classes, with a recall of 92% and an F1-score of 0.85, indicating the model’s strong ability to identify samples from this class with minimal misclassifications correctly. Classes 0 and 4 also performed well, with F1-scores of 0.80 and 0.75, respectively, reflecting a balanced performance in terms of precision and recall. Class 1 achieved a respectable F1-score of 0.72, showing steady performance across metrics. Although Class 3 exhibited the lowest performance, with an F1-score of 0.50, targeted improvements in feature engineering or class-specific data augmentation could enhance its recall and overall classification reliability. The macro average scores (precision: 0.82, recall: 0.84, F1-score: 0.80) underscore the consistent performance across all classes, while the weighted averages (precision: 0.75, recall: 0.76, F1-score: 0.74) reflect the model’s robust handling of class distributions. These results suggest that the model is effective at capturing underlying patterns and distinguishing between most classes, with opportunities for optimisation to reduce misclassifications further and enhance overall performance.

The prominence of cost considerations aligns with behavioural economic models of technology adoption, where perceived financial benefit plays a central role in decision-making. Similarly, environmental motivations correspond to value-based adoption frameworks that emphasise sustainability-oriented identity. The strong preference for proximal charging infrastructure reflects core principles of accessibility theory in transport planning, whereby reduced spatial friction enhances service utilisation. Regional differences observed in cluster dispersion may further be interpreted through spatial equity theory, which highlights how infrastructure concentration and distribution influence mobility behaviour.

This section interprets the findings in relation to the research questions guiding this study as follows:

RQ1: What are the primary demographic, behavioural, and motivational differences between the North East and West Midlands? The clustering and descriptive analyses indicate that cost considerations are more strongly associated with EV adoption motivations in the North East, whereas environmental considerations are relatively more prominent in the West Midlands. Regional income disparities and infrastructure dispersion patterns appear to be associated with behavioural heterogeneity. These findings highlight how socio-economic structure correlates with charging preferences and mobility patterns.

RQ2: How can ML techniques classify EV users based on charging habits and travel patterns? K-Means clustering identified three distinct user segments in each region, demonstrating behavioural stratification in charging frequency, travel tolerance, and infrastructure reliance. The silhouette and Davies-Bouldin indices confirm meaningful cluster separation, particularly in the more homogeneous North East context.

RQ3: What factors are most strongly associated with EV charging preferences? SHAP analysis consistently ranked charging duration, real-time availability, and cost as the most influential features associated with charger selection behaviour. These associations highlight the importance of temporal efficiency and information transparency in shaping infrastructure utilisation.

RQ4: How accurately can predictive models forecast charging behaviour across regions? Predictive performance was stronger in the North East than in the West Midlands, potentially reflecting more homogeneous behavioural patterns. Confidence interval analysis and cross-validation confirm strong predictive capacity within the analysed dataset, while acknowledging generalisation limitations.

RQ5: What strategies can enhance EV infrastructure and support sustainable mobility? Simulation-based optimisation suggests that proximity-sensitive infrastructure placement and improved real-time accessibility may enhance utilisation efficiency. However, such strategies should be interpreted as decision-support insights rather than definitive prescriptions.

### Limitations of the integrated ISE-CAP framework

Several limitations should be acknowledged. First, the dataset consists of 256 respondents, which, although sufficient for structured behavioural modelling and comparative regional analysis, remains modest for complex ML applications. While regularisation and cross-validation techniques were employed to enhance robustness, the relatively small sample size may limit statistical power and increase sensitivity to sample-specific patterns. Second, the study relies on self-reported survey data. Such data may be influenced by recall bias, perception bias, and response subjectivity. Reported charging frequency, waiting tolerance, and behavioural preferences may not perfectly correspond to real-world charging logs or telematics-based behavioural tracking. Future research integrating transactional charging records or longitudinal behavioural datasets would strengthen empirical reliability.

Third, the analysis focuses on two UK regions with distinct socio-economic characteristics. Although this comparative design enhances regional insight, the findings may not be directly generalisable to regions with different infrastructure density, transport networks, climatic conditions, or policy environments. Regional mobility culture and built-environment characteristics may influence EV behaviour in ways not fully captured in this study. Fourth, voluntary participation may introduce selection bias, as individuals who are more engaged with EV communities or sustainability initiatives may be more likely to respond. This may overrepresent early adopters or technologically engaged users. Fifth, although socio-economic variables such as income and education were incorporated into modelling to mitigate confounding effects, residual socio-demographic imbalance between regions may still influence predictive performance and clustering outcomes. The non-probability, self-selection sampling approach limits statistical generalisability beyond the analysed cohort. Findings should therefore be interpreted as behaviourally indicative rather than nationally representative. While the ISE-CAP framework provides a transferable analytical architecture, quantitative policy recommendations derived from this study should be considered region-specific and exploratory. Future research incorporating stratified sampling and national registration datasets would enhance external validity and broader policy generalisation.

Finally, the RL optimisation component was validated within a simulation-based environment rather than through real-world infrastructure deployment. While simulation results demonstrate adaptive optimisation potential, practical implementation would require integration with live grid data, regulatory constraints, and operational feasibility assessments. The present analysis focuses on current EV ownership behaviour and does not explicitly model second-EV adoption intention. Incorporating multi-vehicle ownership dynamics and longitudinal purchase intentions would provide deeper insight into sustained adoption trajectories. These limitations underscore the importance of cautious interpretation and motivate future validation using larger, multi-regional, longitudinal, and real-world operational datasets to enhance external validity and policy applicability. The sample does not constitute a nationally representative EV user dataset. Consequently, national-level policy inferences should be made cautiously, and broader validation using nationally stratified samples is recommended for future research.

## Conclusions

This study presents an advanced analytical framework for EV user behaviour analysis and predictive modelling using the ISE-CAP. This research compares EV adoption, charging preferences, and behavioural patterns across the North East and West Midlands regions by leveraging ML techniques. The clustering analysis reveals three distinct EV user groups in each region, highlighting differences in charging behaviour and adoption drivers. In the North East, cost savings serve as the primary motivation for EV adoption (65%), whereas environmental concerns influence 30% of users in the West Midlands. Predictive models, including RF, CatBoost, and DL architectures, demonstrate high accuracy in forecasting charging behaviours, with the North East model achieving an R$^2$ value of 0.9951 and MSE of 0.0694, compared to the West Midlands model, which attained an R$^2$ value of 0.9548 and MSE of 0.6410. The findings indicate that 85% of users across both regions prefer DC fast chargers, with the majority (66% in the West Midlands and 53% in the North East) willing to travel up to 3 km to access charging stations. The SHAP analysis identifies charging duration, real-time station availability, and cost as the most influential factors in shaping user preferences. To improve EV infrastructure and user satisfaction, this study recommends:

- Expanding the availability of DC fast chargers, particularly in high-demand urban and suburban locations.
- Enhancing real-time accessibility to charging stations through mobile applications and in-car navigation systems.
- Implementing financial incentives to support EV adoption and reduce charging costs, especially for lower-income groups.
- Addressing infrastructure gaps in suburban and rural areas to ensure equitable access to charging facilities.

Policy recommendations may be categorised into phased interventions:

- Short-term interventions include enhancing real-time charger availability information, improving maintenance reliability, and optimising utilisation of existing DC fast chargers.
- Medium-term strategies involve expanding DC fast-charging networks in high-demand corridors, addressing suburban infrastructure gaps, and implementing targeted financial incentives for lower-income groups.
- Long-term interventions focus on integrating adaptive charging optimisation with smart grid systems, incorporating dynamic pricing strategies, and embedding predictive analytics into regional transport planning frameworks.

The application of ISE-CAP demonstrates its effectiveness as a scalable and adaptable methodology for optimising EV infrastructure planning in smart cities. These insights can be a foundation for future research and policy development, ultimately fostering a more sustainable and user-centric EV ecosystem. While the ISE-CAP framework is structurally adaptable and can be applied to other regions with appropriate contextual calibration, its transferability beyond the UK context requires validation using region-specific datasets and infrastructure characteristics. Scalability is contingent upon data availability, behavioural diversity, and policy environments.

## Data Availability

The datasets generated and analysed during the current study, including survey data from both the West Midlands and Newcastle regions, are publicly available in the following GitHub repository: https://github.com/cavusmuhammed68/Scientific-Report. This repository contains raw and preprocessed data in ‘.xlsx‘ format, used for clustering analysis, predictive modelling, and feature importance evaluation.
